# Unfolding the ventral nerve center of chaetognaths

**DOI:** 10.1186/s13064-024-00182-6

**Published:** 2024-05-08

**Authors:** June F. Ordoñez, Tim Wollesen

**Affiliations:** Unit for Integrative Zoology, Department of Evolutionary Biology, University of Vienna, 1030 Vienna, Austria

**Keywords:** Neurogenesis, Mediolateral patterning, In situ hybridization, Gnathifera, Spiralia

## Abstract

**Background:**

Chaetognaths are a clade of marine worm-like invertebrates with a heavily debated phylogenetic position. Their nervous system superficially resembles the protostome type, however, knowledge regarding the molecular processes involved in neurogenesis is lacking. To better understand these processes, we examined the expression profiles of marker genes involved in bilaterian neurogenesis during post-embryonic stages of *Spadella cephaloptera.* We also investigated whether the transcription factor encoding genes involved in neural patterning are regionally expressed in a staggered fashion along the mediolateral axis of the nerve cord as it has been previously demonstrated in selected vertebrate, insect, and annelid models.

**Methods:**

The expression patterns of genes involved in neural differentiation (*elav*), neural patterning (*foxA*, *nkx2.2*, *pax6*, *pax3/7*, and *msx*)*,* and neuronal function (*ChAT* and *VAChT*) were examined in *S. cephaloptera* hatchlings and early juveniles using whole-mount fluorescent in situ hybridization and confocal microscopy.

**Results:**

The *Sce-elav* ^+^ profile of *S. cephaloptera* hatchlings reveals that, within 24 h of post-embryonic development, the developing neural territories are not limited to the regions previously ascribed to the cerebral ganglion, the ventral nerve center (VNC), and the sensory organs, but also extend to previously unreported CNS domains that likely contribute to the ventral cephalic ganglia. In general, the neural patterning genes are expressed in distinct neural subpopulations of the cerebral ganglion and the VNC in hatchlings, eventually becoming broadly expressed with reduced intensity throughout the CNS in early juveniles. Neural patterning gene expression domains are also present outside the CNS, including the digestive tract and sensory organs. *ChAT* and *VAChT* domains within the CNS are predominantly observed in specific subpopulations of the VNC territory adjacent to the ventral longitudinal muscles in hatchlings.

**Conclusions:**

The observed spatial expression domains of bilaterian neural marker gene homologs in *S. cephaloptera* suggest evolutionarily conserved roles in neurogenesis for these genes among bilaterians. Patterning genes expressed in distinct regions of the VNC do not show a staggered medial-to-lateral expression profile directly superimposable to other bilaterian models. Only when the VNC is conceptually laterally unfolded from the longitudinal muscle into a flat structure, an expression pattern bearing resemblance to the proposed conserved bilaterian mediolateral regionalization becomes noticeable. This finding supports the idea of an ancestral mediolateral patterning of the trunk nervous system in bilaterians.

**Supplementary Information:**

The online version contains supplementary material available at 10.1186/s13064-024-00182-6.

## Background

There is a tremendous diversity of body plans in adult and developing bilaterian animals [1]. For a long time, the condensed central nervous systems (CNS) of diverse evolutionary lineages have been considered as an evolutionarily conserved organ system among bilaterians. For the majority of bilaterian clades neuronal condensations in form of glomeruli, ganglia, and brains or nerve cords are well documented, although also organisms with a nerve net as an uncondensed nervous system such as Acoelomorpha do exist [2, 3]. This diversity is also reflected during the ontogeny of these organisms and while embryos may initially possess one or more paired nerve cords, these may have fused secondarily, for example, to a single median nerve cord in adults of some taxa [3]. Interestingly, even in morphologically diverse nervous systems, similar molecular signatures have been observed during nervous system (NS) development [4–6]. These consistently observed similarities in the gene expression profiles during NS development have been proposed as one of the most concrete pieces of evidence of a single evolutionary origin of the bilaterian nerve cord [7–9]. The expression patterns of the transcription factor encoding genes *foxA*, *nkx2.1*/*nkx2.2*, *nkx6*, *pax6*, *pax3/7*, and *msx* have shown similar staggered mediolateral patterns in the developing nerve cord of some bilaterian representatives, including vertebrates, the fruit fly *Drosophila melanogaster*, and the annelid *Platynereis dumerilii* [reviewed in 2, 4]. In addition, the spatial organization of their expression patterns corresponds to the mediolateral regionalization of different neuronal cell subtypes along the neural midline of the developing CNS. Serotonergic neurons form along the medial domain (*nk2.2* ^+^ */nk6* ^+^), cholinergic neurons and interneurons at the intermediate domain (*pax6* ^+^ */nk6* ^+^ and *pax6* ^+^ */pax3/7* ^+^, respectively), and sensory neurons at the lateral domain (*msx* ^+^ */pax3/7* ^+^) [5, 6, 8, 9]. The mediolaterally staggered expression patterns in these neuronal cell populations have been a pivotal basis for proposing the hypothesis of a medially condensed ventral nerve cord that features a mediolateral patterning system in the bilaterian ancestor [4–7, 9]. However, this mediolateral patterning has been argued to be absent in the annelid *Owenia fusiformis*, the rotifer *Epiphanes senta*, the nemertean *Lineus ruber* [10], the nematode *Caenorhabditis elegans* [11], onychophorans [12–14], and the hemichordate enteropneust *Saccoglossus kowalevskii* [15–17]. In addition, in several other phylogenetically informative taxa, including acoelomorphs and brachiopods, mediolateral patterning has not been (or only partially) observed [5, 10]. The lack of mediolateral patterning in the latter taxa has led to the argument that nerve cords evolved independently in several bilaterian lineages ([10, 18] but see [5] for an alternative standpoint). Additional arguments against the evolutionary conservation of nerve cords among Bilateria are the divergent ontogenetic histories of the latter in phylogenetically distantly related taxa such as annelids, vertebrates, or arthropods. While the early neuroectoderm of e.g. hemichordates and chordates undergoes an infolding process into a tube [19], among panarthropods neuroblasts delaminate from the neuroectoderm in the region giving rise to the head and the ventral nerve cords [20]. Subsequent fates of these neuroblasts may, however, differ considerably. In spiralians such as annelids or mollusks, early neuroectoderm invaginates and forms nerve cords and ganglia of varying numbers and of different organizations in different representatives [3].

Although the currently available data either substantiate or refute the hypothesis that mediolateral patterning during CNS development is conserved in Bilateria, our understanding of the ancestral states of neuroectoderm mediolateral patterning and the origin of nerve cords remains incomplete. This is principally due to the lack of information on the molecular architecture of key bilaterian groups, especially those that exhibit unique CNS features and represent an evolutionarily relevant taxonomic branch. Therefore, further investigation into additional bilaterian clades and developmental stages is imperative to infer the evolutionary history of the CNS.

**Fig. 1:**
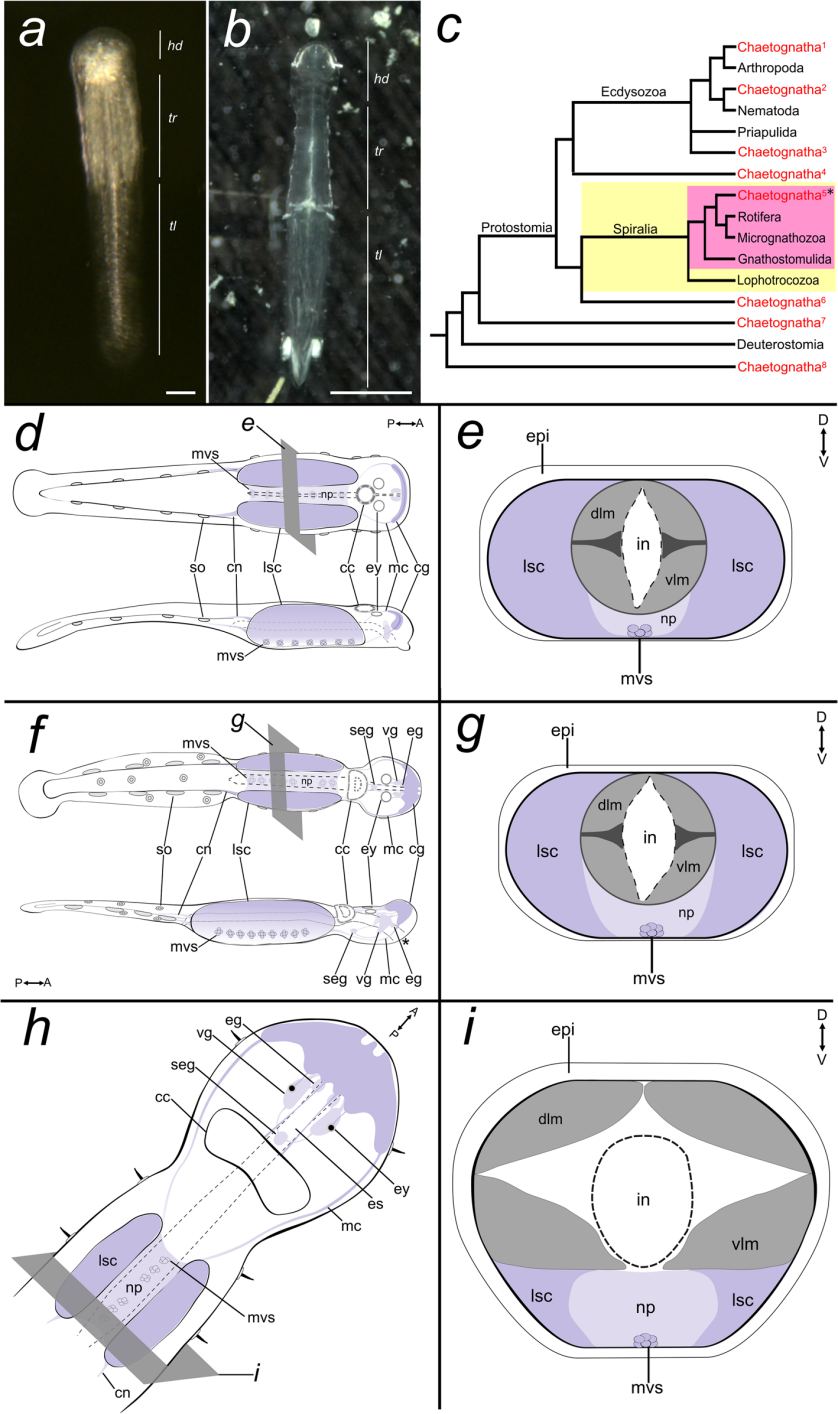
General architecture of the chaetognath nervous system. **a** A hatchling and an adult (**b**) *Spadella cephaloptera*. **c** Possible phylogenetic positions of the Chaetognatha based on various molecular studies (modified from [23]), with position five having the highest number of supporting studies (asterisk). **d**, **f**, **h** General architecture of the nervous system of chaetognath hatchling (**d**), young juvenile (**f**), and adult (**h**) (modified from [21, 25]). Neural regions (in purple) of the hatchling are based on immunostaining analyses of Rieger et al. [25] and *Sce-elav* expression pattern (this study). **e**, **g** Transverse section of the trunk of hatchling, young juvenile, and adult. In panels *d* – *i*, the intestine is indicated by dashed outline. The asterisk indicates the position of the mouth. 1: Zrzavý et al. (1998) [27]; 2: Halanych (1996) [28], Peterson & Eernisse (2001) [29]; 3: Paps et al. (2009) [30], Marlétaz et al. 2019 [26]; 4: Mallat & Winchell (2002) [31]; 5: Littlewood et al. (1998) [32]; 6: Matus et al. (2006) [33], Papillon et al. (2004) [34], Dunn et al. (2008) [35], Philippe et al. (2011) [36]; 7: Giribet et al. (2000) [37], Helfenbein et al. (2004) [38], Marlétaz et al. (2006) [39]; 8: Telford & Holland (1993) [40]; Papillon et al. (2003) [41]; cc, corona ciliata; cg, cerebral ganglion; cn: caudal nerve; dlm, dorsal longitudinal muscle; eg, esophageal ganglion; es, esophagus; epi: epidermis; ey: eye; hd: head; in, instestine; lsc, lateral somata clusters; mc: main connective; mvs, medioventral somata clusters; np, trunk neuropil; seg, subesophageal ganglion; so, ciliary sensory organ; tl: tail; tr: trunk; vlm, ventral longitudinal muscle, vsg, vestibular ganglion. Scale bars equal 50 µm (**a**) and 130 µm (**b**)

Chaetognatha (arrow worms) is one of the animal groups that exhibit interesting NS anatomy as well as a phylogenetically informative position (Fig. 1a, b). They are small invertebrate predators that constitute a major portion of the global ocean zooplankton [21]. Arrow worms exhibit genetic and embryonic developmental features that resemble those of protostomes and deuterostomes, resulting in varying phylogenetic positions within Bilateria (Fig. 1c; reviewed in [22–25]). However, based on a comparison of some bilaterian genome-wide transcriptomes, chaetognaths have recently been suggested to belong to the clade Gnathifera (Ahlrichs, 1995), which includes animals with internal chitinous jaw-like structures such as gnathostomulids (jaw worms) and rotifers (wheel animals) [26].

Little is also known about early chaetognath neurogenesis. In *S. cephaloptera*, the neural progenitors that give rise to the VNC have been first observed in the anterolateral regions of the ectoderm during early embryogenesis (Fig. 7 d) [42]. These neurogenic cells multiply their nuclei and the latter eventually sink inwards and form the progenitor cells of the VNC (referred to as ganglion cells by [42]). Subsequently, these ganglion cells separate from the row of ectodermal cells, aggregate into a mass of cells in the ventrolateral region of the mesodermal cell bands (future longitudinal muscle), and successively form two lateral somata clusters as apparent in hatchlings (Fig. 1e).

The nervous system of the chaetognath hatchling resembles that of the adult to some extent (Fig. 1a, b, d, h). Both possess a dorsal cerebral ganglion that is connected to a ventrally positioned ganglion (VNC) along the trunk (Fig. 1d, e, h, i) [23, 25, 43]. As the hatchling matures, the rudimentary loop-like cephalic ganglion becomes structurally more complex and develops additional cephalic ganglia, including the vestibular and esophageal ganglia. The VNC features a central neuropil bordered by two bands of somata clusters on the lateral sides (lateral somata clusters) and small clusters of cells on its medioventral side (medioventral somata clusters) [23, 25]. The VNC is already well established at hatching [25]. While it wraps around the centrally positioned muscle and gut bundle in hatchlings (Fig. 1e), it eventually becomes restricted to the midventral region underneath the longitudinal muscle bands of the adult trunk (Fig. 1g) [23, 25, 43]. Chaetognaths display typical protostomal neuroarchitecture such as the circumoral nervous system; however, the adult brain exhibits two components (i.e. anterior and posterior brain) that form two circumoral nerve rings connected to the ventral ganglia of the head and the trunk [25, 43]. In addition, arrow worms also possess a number of unique neural structures, organizational patterns, and developmental features that are remarkably different from other gnathiferans. For example, they possess a neuro-muscular boundary that lacks specialized junctions of axonal varicosities, pre-synapses and the underlying muscles and is separated by a thick extracellular matrix [23, 25]. They also feature an intraepidermal ventral nerve center [25], and other apomorphic traits known from early neurogenesis which do not appear to be closely related to traits of either deuterostomes or other protostomes [25].

So far, the morphology of the chaetognath nervous system has been adequately described [43–46] and there have been comparative morphological analyses of neural features (e.g. ventral nerve cord) between chaetognaths and other taxa, such as annelids and arthropods [10–12]. However, our understanding of their neurogenesis and neural patterning, especially the molecular regulatory circuitry, underlying these processes, remains incomplete.

To further explore the molecular aspects of nervous system development in chaetognaths, we performed an in-depth characterization of evolutionarily highly conserved genes involved in bilaterian neurogenesis in the early post-embryonic stages of *Spadella cephaloptera*. We determined whether these axial patterning genes are regionally expressed along the mediolateral axis of the developing nerve cord similar to what is observed in diverse bilaterian representatives identified in [16, 26]. We first screened for gene orthologs of *foxA*, *nkx2.2*, *pax6*, *pax3/7*, *msx*, *ChAT*, *VAChT*, and *elav* in the draft transcriptome of *S. cephaloptera* [47] and examined their spatial expression using *in situ* hybridization on hatchlings and early juveniles. By comparing our findings with data from other bilaterians, we were able to identify similarities in the expression patterns of these gene markers in the CNS. Ultimately, this provided insights into the putative roles of these genes during late neural development of chaetognaths. All the above-mentioned genes play important roles in early neural patterning, a process that this study could not document due to the unsuccessful *in situ* hybridization experiments on earlier encapsulated chaetognath embryos. Nevertheless, our findings show that these transcription factors probably play an evolutionarily conserved role during the formation of the CNS in chaetognath hatchlings, similar to other bilaterians. At first glance, chaetognath mediolateral gene expression patterns appear partially scrambled compared to those of other bilaterian models. However, in chaetognath hatchlings, the conceptual unfolding of the VNC resembles the postulated ancestral bilaterian staggered array, thus reinforcing the view that this mediolateral array is part of a key ancestral process during the formation of the trunk nervous system in bilaterians.

## Methods

### Animal collection, husbandry, and fixation

Live adult specimens of *Spadella cephaloptera* (Busch, 1851) were collected in the intertidal zone off the shore of Roscoff, France, and maintained in aquaria with a blend of artificial and natural sea water (kept at 18°C and 35 ppt) in the aquatic research facility of the University of Vienna. Mature individuals (n ~ 35) were collected from tanks and transferred to culture plates (14.1 cm radius Petri dishes) and fed with *Artemia sp.* nauplii until egg-laying. One-day post-hatching individuals (8 – 20h old hatchlings; 1 dph) and early juveniles (7 – 10 dph) were manually collected from the culture plates and fixed in MEM-PFA (0.5 M MOPS, 5 mM EGTA, 10 mM MgSO_4_, 2.5M NaCl, and 4% paraformaldehyde) for one hour at room temperature. They were washed three times for five minutes each in PBTw (1x PBS, pH 7.5 with 0.1% Tween-20), followed by three 15-minute washes with 100% methanol, and were then stored at –20°C in methanol. *In situ* hybridization experiments have been carried out on encapsulated prehatching embryos without success because it was not possible to remove or open the egg capsule without destroying the embryo. Consequently, this study only focused on early post-embryonic stages.

### Gene identification and orthology assignment

Candidate genes (*msx*, *pax3/7*, *pax6*, *nkx2.2*, *foxA*, *ChAT*, *VAChT*, and *elav*) were identified in the *S. cephaloptera* draft transcriptome [47] using blastx [48] searches against protein sequences from NCBI Genbank. Phylogenetic reconstruction was used to determine gene orthology. Representative homologous amino acid sequences from other bilaterians were downloaded from NCBI Genbank. Multiple sequence alignments were performed with ClustalW [49] implemented in Geneious Prime (v2023.0.1) and were subsequently trimmed with trimai (v1.2) [50]. For each alignment, Prottest 3 (v3.4.2) [51] was used to determine the most suitable amino-acid substitution model based on AIC score. Bayesian phylogenetic analyses were conducted with MrBayes (v3.2.6) [52] plugin in Geneious Prime. Accession numbers and substitution models used are provided in Additional Table 1. *S. cephaloptera* gene sequences were uploaded to GenBank Database with accession numbers: OR701369-OR701376.

### Sequencing and riboprobe synthesis

Different developmental stages of *S. cephaloptera* were sacrificed and pooled for total RNA extraction using RNAqueous™-Micro Total RNA Isolation Kit (Invitrogen GmbH, Karlsruhe, Germany), which was then used for cDNA synthesis using First Strand cDNA Synthesis Kit for RT-PCR (AMV) (Roche Molecular Biochemicals, Vienna, Austria).

Primers were designed for the identified transcripts using Geneious Prime or Primer-BLAST (https://www.ncbi.nlm.nih.gov/tools/primer-blast/). Reverse primers were synthesized with a T7 promoter sequence (5′-TAATACGACTCACTATAGGG-3′) at the 5’ end. To generate the DNA template for the riboprobe, PCR experiments were performed using these gene-specific primers and Taq 2X Master Mix (New England Biolabs). PCR amplicons were purified with QIAquick PCR Purification Kit (Qiagen Vertriebs GmbH, Vienna, Austria) and were sent to Microsynth Austria for Sanger sequencing to validate their identity. The resulting electropherograms were visually inspected with Geneious Prime. Digoxigenin (DIG)-labeled antisense riboprobes were generated using the T7 polymerase and DIG RNA labeling mix kit (Roche Diagnostics, Mannheim, Germany). Total riboprobe concentrations were estimated using a NanoDrop 2000 spectrophotometer (ThermoFisher Scientific, Waltham, MA). All primer sequences and their annealing temperatures are available in Additional file 2.

### Whole-mount fluorescent in situ hybridization

WM-FISH experiments on early post-embryonic stages (1 dph and 7 – 10 dph) were carried out following the protocol of Wollesen [47] with some modifications: Specimens were digested with 2 µg/mL Proteinase K (Roche Diagnostics, Mannheim, Germany) diluted in PBS with 0.1% Tween-20 at 37˚C for 15 minutes. Hybridization was performed with overnight water-bath incubation at 61°C (18 – 22h). Color reaction was visualized in 0.25 mg/mL Fast Blue BB and 0.25 mg/mL NAMP (Sigma Chemical Co., St. Louis, Missouri, USA) in SB8.2 (0.1 M Tris-HCl pH 8.2, containing 50 mM MgCl_2_, 100 mM NaCl, 0.1% Tween-20) as previously described by [53].

### Microscopy and image processing

Samples were imaged on a Leica TCS SP5 confocal microscope (Leica Microsystems, Heidelberg, Germany). A 633 nm gas laser was used for the fluorescent scanning of Fast Blue stains and the 405 nm laser was used to visualize the DAPI-stained perikarya. Fiji [54] was used to view and adjust confocal images for brightness and contrast and to perform z-stack projections of the optical sections. IMARIS (v 3.4.1) was also used for 3D visualization and to capture images of the orthogonal sections (lateral and transverse planes). Figure assembly and schematic drawings were prepared with Inkscape (https://inkscape.org). All anatomical identifications used here were based on the descriptions of [23–25, 55–57].

## Results

### Gene orthologs and phylogenetic analyses

Putative orthologs of *foxA*, *nkx2.2*, *pax6*, *pax3/7*, *msx*, *ChAT*, *VAChT*, and *elav* were identified in the draft transcriptome of *S. cephaloptera*. Only *Sce-chat*, *Sce-nkx2.2* and *Sce-msx* showed an incomplete open reading frame, while the remaining genes were full-length. Phylogenetic analyses based on a Bayesian approach confirmed the orthology of *S. cephaloptera* sequences to their bilaterian orthologs (see Additional file 3).

#### Neural regions of the *Spadella* hatchling

**Fig. 2:**
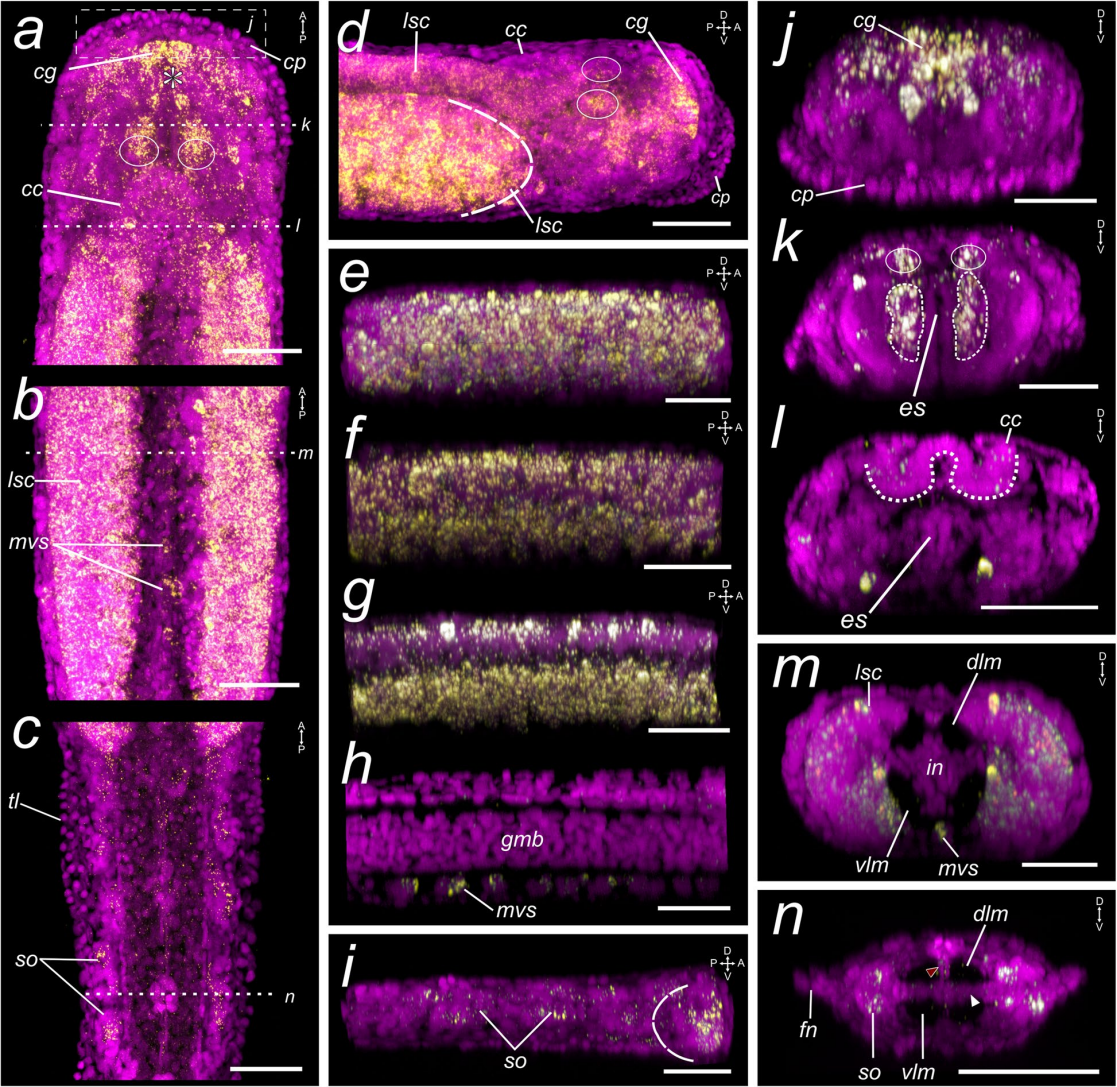
Expression pattern of *elav* in the *Spadella cephaloptera* hatchling (1 dph). Gene transcripts are visualized with AP-Fast Blue (yellow) and cell nuclei with DAPI (purple). Maximum intensity projection of the (**a**) head, (**b**) trunk, and (**c**) tail. **d** Lateral projection of the head. Eyes are encircled and the dashed curve demarcates the anterior border of the lateral somata clusters. **e**–**h** Lateral projections of the trunk from the lateral region (**e**) to the (**h**) longitudinal midline. **g** Lateral section along the border of the gut-muscle bundle and the lateral somata cluster. **i** Lateral projection of a tail section. **j**–**l** Transverse sections of head showing the *Sce-elav* expression in (**j**) the cerebral ganglion, (**k**) the eyes (circle) and the cell clusters (with dashed borders) lateral to the esophagus and (**l**) slightly in the corona ciliata. Transverse sections of the (**m**) trunk and (**n**) tail. The location of the transverse section is indicated in panels a – c. Scale bars: 50 μm. The asterisk indicates the position of the future mouth opening. Orientation of specimens is indicated in the top right corner of each panel. cc, corona ciliata; cg, cerebral ganglion; cp, cephalic adhesive papillae; dlm, dorsal longitudinal muscle; es, esophagus; fn, fin; gmb, gut-muscle bundle; lsc, lateral somata clusters; mvs, medioventral somata clusters; so, ciliary sensory organ; tl, tail; vlm, ventral longitudinal muscle

To further describe the neural domains of the *Spadella* hatchlings, we examined the expression patterns of the marker for postmitotic neuronal precursor cells in many bilaterians [58, 59]. In the head of hatchlings, *Sce-elav* transcripts are detected in the cells of the developing brain (Fig. 2a, d, j), in a pair of somata clusters bordering the esophagus (presumptive developing vestibular ganglia) (Fig. 2k), in the eyes (Fig. 2a, d, k, circles), and at a low level in cells of the corona ciliata (Fig. 2a, l, dashed outline). In the trunk, *Sce-elav* expression is observed in cells of the lateral and medioventral somata clusters (Fig. 2b, e – h, m). The expression in the tail is detected in cells of lateral ciliary sense organs (Fig. 2c, i, h).

Early juveniles exhibit *Sce-elav* expression in the cerebral ganglion (Additional file 4: Fig. S8a, e, f, k, l), the vestibular ganglia (Additional file 4: Fig. S8b, g, k), the esophageal ganglia (Additional file 4: Fig. S8b, c, arrowheads), the presumptive subesophageal ganglion (Additional file 4: Fig. S8d, i, l), and to a lesser degree, in the eyes and the corona ciliata (Additional file 4: Fig. S8a, j-l). Transcripts are also present in the lateral and medioventral somata clusters (Additional file 4: Fig. S8m, n, p, q). In the tail, *Sce-elav* is expressed in cells situated at the trunk-tail border where the germ cells are positioned (Additional file 4: Fig. S8n, r, t, arrowheads), in lateral cells posterior to it (Additional file 4: Fig. S8r, arrows), and in the ciliary tuft organs and fence receptors (Additional file 4: Fig. S8r – t).

### Expression of neural mediolateral patterning genes in *Spadella*

The expression patterns of mediolateral patterning genes in hatchlings and juveniles of *S. cephaloptera* were determined using fluorescent whole-mount *in situ* hybridization (WMFISH). Dorsal scans are presented as z-stack projections, while lateral and transverse sections are shown as one optical section. All additional figures in the succeeding sections have been deposited in the Supplementary material file.

### Expression of *Sce-foxA*

**Fig. 3:**
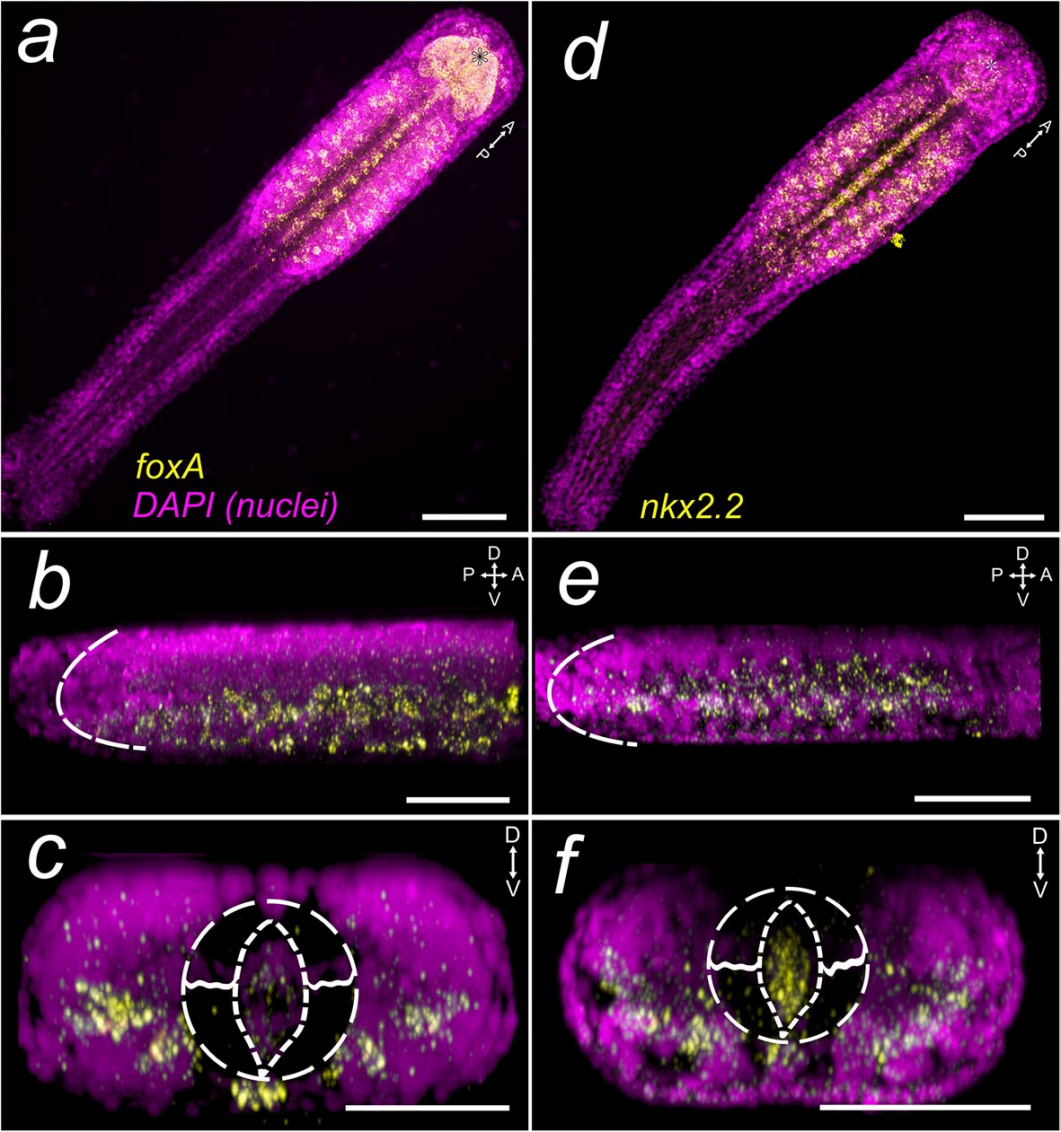
Expression patterns of *Sce-foxA* and *Sce-nkx2.2* in the *Spadella cephaloptera* hatchling (1 dph). Gene transcripts are visualized with AP-Fast Blue (yellow) and cell nuclei with DAPI (purple). **a**-**c** *Sce-foxA,* (**d**-**f**) *Sce-nkx2.2.* **a**, **d** Horizontal projection of the entire hatchling. **b**, **e** Lateral projection showing the expression pattern in the VNC. Dashed curves demarcate the posterior end of the VNC. **c**, **f** Transverse projection of the VNC. The dashed circle indicates the region of the gut (almond-shaped center)—longitudinal muscle bundle. Scale bars: 50 μm, except for panels a, d (100 μm). The asterisk indicates the position of the mouth opening. Orientation of specimens is indicated in the top right corner of each panel. See Fig. 1 for morphological description

A large region of the head shows strong *Sce-foxA* expression: the mesodermal-derived cells of the developing pharynx and esophagus; a bulbous-shaped structure, resembling the perioral epidermis in adults, that seemingly envelopes the inner regions of the head where most of the cephalic muscles develop (Fig. 3a; Additional file 5: Fig. S9a, b, e-g, red arrowheads); and a pair of cell clusters (Fig. 3a; Additional file 5: Fig. S9b, e, dashed oval), which presumably corresponds to the developing vestibular and esophageal ganglia, laterally flanking the esophagus (solid lines). In addition, *Sce-foxA* ^+^ cells are also located in the ventral-most region of the head and posterior to the future buccal cavity (Additional file 5: Fig. S9c). In the trunk, *Sce-foxA* is expressed in the medioventral neurons (Fig. 3a, c; Additional file 5: Fig. S9i’, k) and in distinct lateral neuronal cells that are arranged in two rows parallel to the gut: a more lateral domain (Fig. 3c; Additional file 5: Fig. S9i) and a ventral domain proximal to the midline, slightly dorsal to the medioventral neuronal cells (Additional file 5: Fig. S9i’). The more lateral rows (Additional file 5: Fig. S9i) include large cells that feature an inconspicuously large nucleus interspersed within the smaller neuronal cells (Additional file 5: Fig. S9 j, j’). The gene transcripts are also distinguished along the entire trunk portion of the gut (Additional file 5: Fig. S9i, arrows, k) and in a group of cells medioposterior to the somata of the ventral nerve center (Additional file 5: Fig. S9i’, l, arrowhead). In early juveniles, *Sce-foxA* is evidently expressed in the anterior-most organs of the head, including the cerebral ganglion (Fig. 6a-c; Additional file 5: Fig. S9m, q, s, t), the vestibular ganglia (Additional file 5: Fig. S9n, r, dashed outlines), and the cells of the structure surrounding the buccal cavity (more specifically perioral epidermis) where the teeth develop (Fig. 6a-c; Additional file 5: Fig. S9n – r). *FoxA* expression is also detected in cells of the lateral plates (Additional file 5: Fig. S9o) and the ventral-most cells of the head, posterior to the mouth opening (Additional file 5: Fig. S9p). In the VNC, *Sce-foxA* is expressed in the medioventral neurons and, unlike in hatchlings, the expression increases in the lateral somata cluster (Fig. 6a-c). No expression is observed in the tail except for the ventral cells medially situated on the posterior border of the VNC, where the presumptive anal pore is positioned (Additional file 5: Fig. S9u-w, arrowhead).

### Expression of *Sce-nkx2.2*

In the neural domains, *Sce-nkx2.2* is expressed in cells of the lateral somata clusters and is less pronounced in the medioventral somata clusters (Fig. 3d-f; Additional file 6: Fig. S10h, i). Low *Sce-nkx2.2* expression was observed in the anterior head region where the cerebral ganglion is developing (Additional file 6: Fig. S10a, d, g). A pair of cell clusters lateral to the oral cavity is also *Sce-nkx2.2* positive (Additional file 6: Fig. S10a, b, d, white arrowheads). Additionally, the expression is prominent along the digestive tract, spanning from the esophageal region (posterior to the future mouth) and extending throughout the entire intestine (Fig. 3d; Additional file 6: Fig. S10b, f, g, i). *Sce-nkx2.2* is also detected in the ventral cells situated on the trunk-tail boundary (Additional file 6: Fig. S10h, i, arrowhead). No *Sce-nkx2.2* signal was observed in the early juvenile (data not shown).

### Expression of *Sce-pax6*

**Fig. 4:**
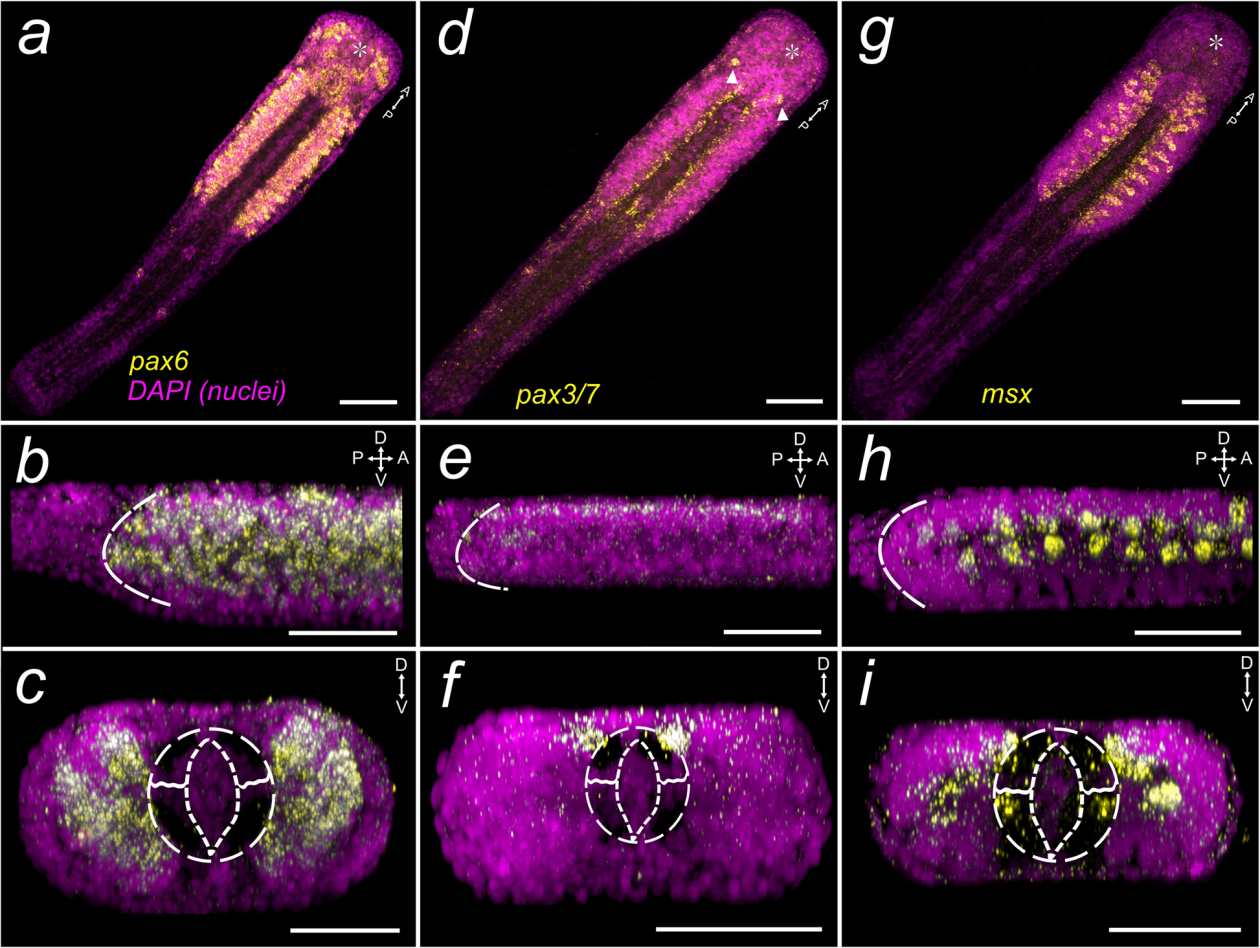
*Sce-pax3/7*, *Sce-pax6*, and *Sce-msx* expression patterns in the hatchling of *S. cephaloptera* (1 dph). Gene transcripts are visualized with AP-Fast Blue (yellow) and cell nuclei with DAPI (purple). **a**–**c ***Sce-pax6,* (**d**–**f**) Sce-pax3/7*,* (**g**–**i**) *Sce-msx*. **a**, **d**, **g** Horizontal projection of the entire hatchling. (d) *Sce-pax3/7* ^+^ neurons in the anterior VNC (arrowheads). **b**, **e**, **h** Lateral projection showing the expression pattern in the VNC. Dashed curves demarcate the posterior end of the VNC. **c**, **f**, **i** transverse projection of the VNC. The dashed circle indicates the region of the gut (almond-shaped center)—longitudinal muscle bundle. Scale bars: 50 μm, except panels (**a**, **d**, **g**;100 μm). The asterisk indicates the position of the mouth opening. Orientation of specimens is indicated in the top right corner of each panel. See Fig. 1 for morphological description

*Sce-pax6* is expressed in the dorsal anterolateral portion of the head (Fig. 4a; Additional file 8: Fig. S11a, c, f). Transcripts were also detected in sensory structures between the two concentric rings of the corona ciliata, the eyes, and the lateral sensory organs of the trunk and tail (Fig. 4a; Additional file 7: Fig. S11a, d, h, i). In the trunk, *Sce-pax6* is expressed in cells of the lateral somata clusters (Fig. 4a – c; Additional file 7: Fig. S11a, b, g) but not in the mediodorsal region of the VNC (Fig. 4b). Similar expression domains are generally observed in early juveniles (Fig. 6 g-i; Additional file 7: Fig. S11j–s) except for a weaker signal in the eyes and corona ciliata (Additional file 7: Fig. S11j, o, p). *Sce-pax6* is still evident in the anterolateral portions of cephalic ganglia (Fig. 6g; Additional file 7: Fig. S11j). In addition, *Sce-pax6* is strongly expressed in the anterior head (presumably the anterior-most part of the brain flanked by a pair of frontal connectives (Additional file 7: Fig. S11m), the posterior brain adjacent to the retrocerebral organ (Additional file 7: Fig. S11j), in cells of the presumptive esophageal ganglia (Additional file 7: Fig. S11k, n, arrowheads), in cells lining the buccal area (presumably perioral epidermis; Additional file 7: Fig. S11l, o), and in medioventral neuronal cells (Additional file 7: Fig. S11q’). In the tail, a few ciliary fence organs also express *Sce-pax6* (Additional file 7: Fig. S11r, s), although slightly less intense than in 1 dph individuals.

### Expression of *Sce-pax3/7*

*Sce-pax3/7* is expressed in a pair of cells with large cell nuclei in the anteroventral tip of the lateral somata clusters of the hatchling (Fig. 4e, Additional file 8: Fig. S12a, b. b’). In the VNC, the *Sce-pax3/7* expression domain appears in two narrow longitudinal stripes that follow the contour of the entire inner side contour of the lateral somata clusters that flank the dorsal longitudinal muscles (Fig 4d-f; Additional file 8: Fig. S12c, f), in presumptive mesodermal cells on both sides of the developing germ cells (Additional file 8: Fig. S12 c, d, g, h, arrow and arrowheads), and in cells situated on the ventral anterior tail, which could be the position for the future anus (Additional file 8: Fig. S12e – g, red arrowhead). Early juveniles exhibit Sce-*pax3/7* expression in neural regions of the head, including the brain, vestibular ganglia, and esophageal ganglia (Fig. 6d; Additional file 8: Fig. S12i – k). In the lateral somata clusters, the expression becomes diffused (Fig. 6d-f; Additional file 8: Fig. S12k). *Sce-pax3/7* is also detected in the presumptive specialized mesodermal cells comprising the posterior septum (Additional file 8: Fig. S12m – p) and in cells around the anal region (Additional file 8: Fig. S12l, n, p).

### Expression of *Sce-msx*

*Sce-msx* expression domains in the head of the *Spadella* hatchling are observed in the dorsal cells slightly anterior to the eyes (Fig. 4g, h; Additional file 9: Fig. S13a – c, arrowheads) and in the ventral cells situated between corona ciliata and the eyes (Fig. 4g, h; Additional file 9: Fig. S13a, d, arrows)*. Sce-msx* is largely localized in the medio-dorsal region of the lateral somata clusters (Fig. 4g – i; Additional file 9: Fig. S13e, f, f’). This includes some distinguishable large *Sce-msx*^+^ neuronal cells that are dorsally arranged in rows. These cells occupy a larger portion of the lateral somata cluster and appear to possess less distinct nuclei compared to the smaller surrounding cells (Additional file 9: Fig. S13g, g’). In addition, *Sce-msx* transcripts are also present in the longitudinal muscle of the trunk, but more distinctly in the ventral longitudinal muscles (Fig. 4i; Additional file 9: Fig. S13f’, arrowhead). Early juveniles exhibit *Sce*-*msx* expression in the brain and the vestibular and esophageal ganglia (Fig. 6j; Additional file 9: Fig. S13h, i). The lateral somata clusters have diffused *Sce*-*msx* expression (Fig. 6j; Additional file 9: Fig. S13h – j, j’). In addition, *Sce-msx* appears to be absent in the longitudinal muscles of early juveniles.

### Expression of the cholinergic markers *Sce-ChAT* and *Sce-VAChT*

**Fig. 5:**
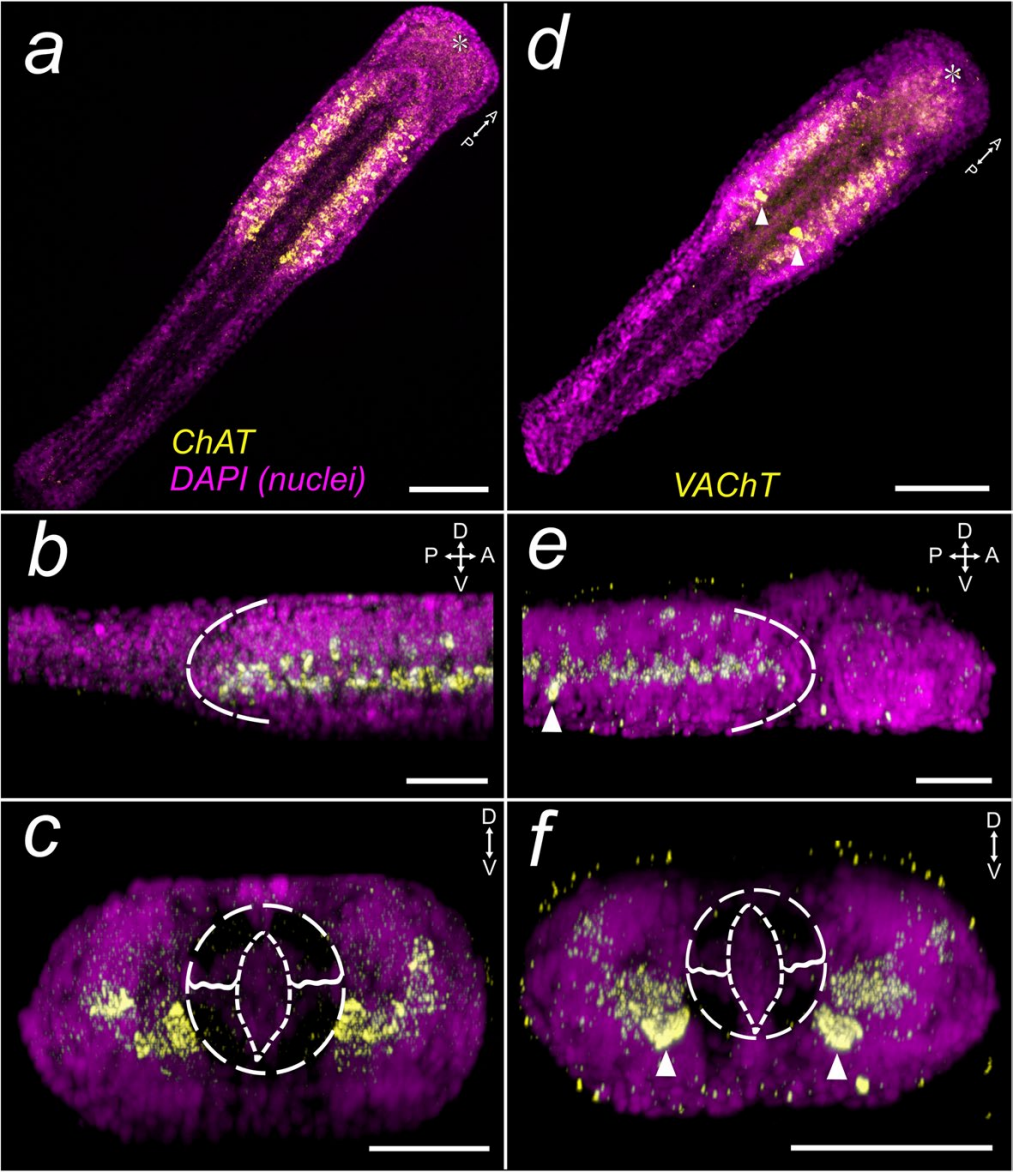
Expression patterns of cholinergic markers in the hatchling of *Spadella cephaloptera* (1 dph). Gene transcripts are visualized with AP-Fast Blue (yellow) and nuclei with DAPI (purple). **a-c** *Sce-ChAT,* (**d-f**) *Sce-VAChT.* **a**, **d** Horizontal projection of the entire hatchling. **b**, **e** Lateral projection showing the expression patterns in the VNC. Dashed curves demarcate the posterior (**b**) or anterior (**e**) end of the VNC. **c**, **f** Transverse projection of the VNC. **e**, **f** Overexpression of *Sce-VAChT* is observed in two large bilateral neurons in the posterior VNC (arrowheads). The dashed circle indicates the region of the gut (almond-shaped center)—longitudinal muscle bundle. Scale bars: 50 μm, except panels a, d (100 μm). The asterisk indicates the position of the mouth opening. Orientation of specimens is indicated in the top right corner of each panel. See Fig. 1 for morphological description

**Fig. 6:**
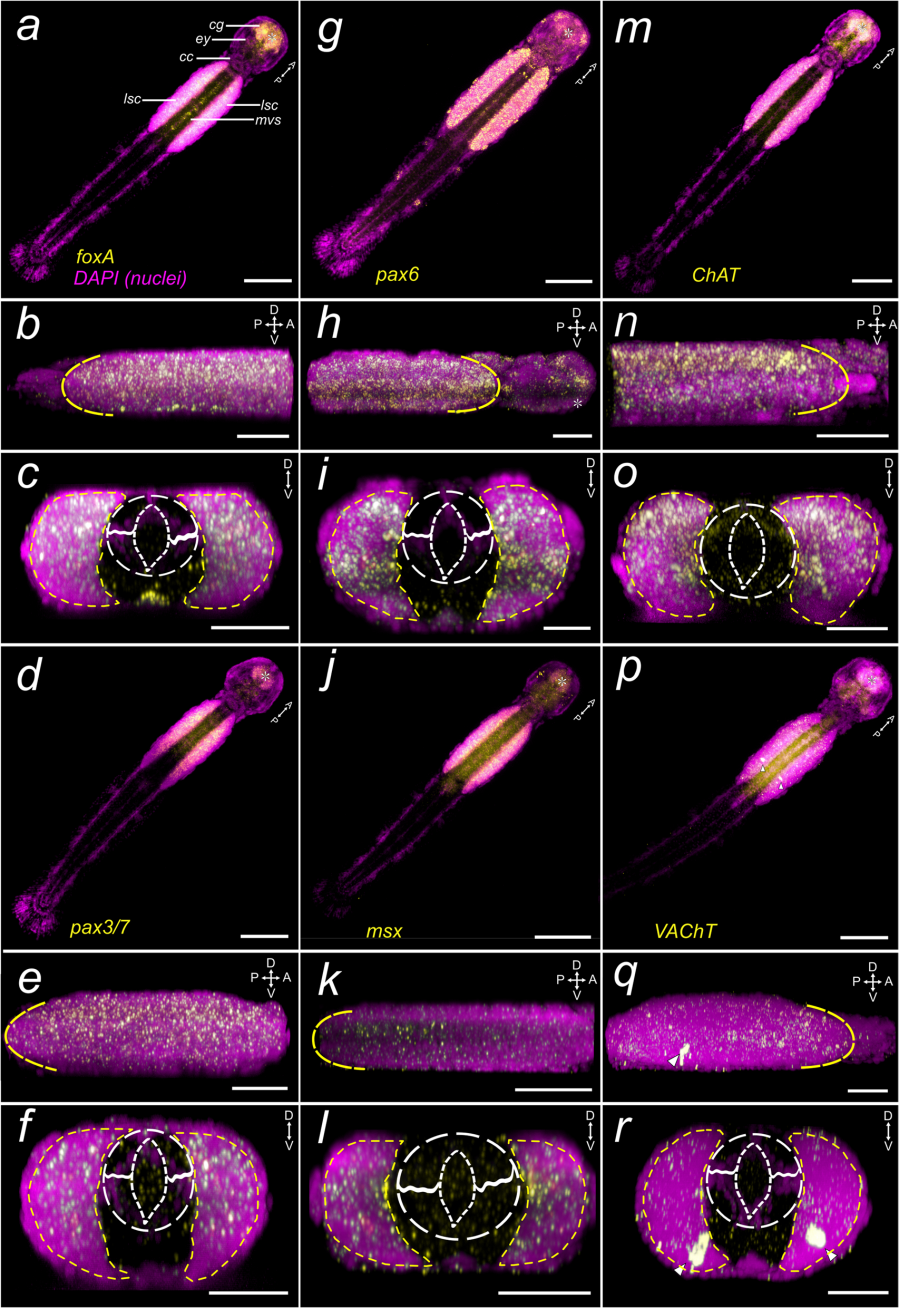
Expression of CNS patterning markers in the early *Spadella cephaloptera* juvenile. Gene transcripts are visualized with AP-Fast Blue (yellow) and cell nuclei with DAPI (purple). **a**-**c** *Sce-foxA*, (**d**-**f**) *Sce-pax3/7*, (**g**-**i**) *Sce-pax6*, (**j**-**l**) *Sce-msx*, (**m**–**o**) *Sce-ChAT*, and (**p**-**r**) *Sce-VAChT*. **a**, **d**, **g**, **j**, **m**, **p** Horizontal projections of entire animals (scale bar: 100 µm). **b**, **e**, **h**, **k**, **n**, **q** Lateral projections showing the expression patterns in the VNC (scale bar: 50 µm). Dashed curves demarcate the posterior (**b**, **e**, **k**) or anterior (**h**, **n**, **q**) end of the VNC. **c**, **f**, **i**, **l**, **o**, **r** show transverse projection of the VNC with lsc in yellow dashes. **p**, **q**, **r** Strong expression of *Sce-VAChT* is observed in two, large, bilateral neurons in the posterior VNC (arrowheads). The dashed circle indicates the region of the gut (almond-shaped center)—longitudinal muscle bundle. Scale bars: 50 μm, except panels **a**, **d**, **g**, **j**, **m**, and **p** (100 μm). The asterisk indicates the position of the mouth opening. Orientation of specimens is indicated in the top right corner of each panel. cc, corona ciliata; cg, cerebral ganglion; ey: eye; lsc, lateral somata clusters; mvs, medioventral somata clusters

The expression of *Sce-ChAT* and *Sce-VAChT* appears to be predominant in the medial (inner) cells of the lateral somata clusters, abutting the medial anterior neuropil and ventral longitudinal muscle (Fig. 5; Additional file 10: Fig. S14a, b; Additional file 11: Fig. S15a, b). Several *Sce-ChAT* ^+^ cells in the dorsolateral portion of the clusters are also identifiable in the hatchling (Additional file 10: Fig. S14a-c). In addition, *Sce-ChAT* is observed in encapsulated late embryos, where it is expressed in the medial lateral somata cluster (Additional file 10: Fig. S14d). *Sce-VAChT* expression is also present in cells lateral to the buccal opening (Additional file 11: Fig. S15b, c, arrowheads). *Sce-ChAT* and *Sce-VAChT* expression domains in early juveniles (Fig. 6m-r) are both observed in the cerebral ganglion (Fig. 6 m, p; Additional file 10: Fig. S14, e, i, m, n). In the lateral somata clusters, *Sce-VAChT* exhibits diffuse expression (Fig. 6p – r; Additional file 11: Fig. S15e), while *Sce-ChAT* expression is detected at a higher-intensity in the dorsal portion (Fig. 6o; Additional file 10: Fig. S14m). Notably, a pair of somata exhibiting high expression intensity of *Sce-VAChT* is also observed in the dorso-posterior region of the lateral somata clusters in 1 dph (Fig. 5d – f; Additional file 10: Fig. S14a, d, arrowheads, f) which persists in early juveniles (Fig. 6p – r; Additional file 11: Fig. S15e, g).

## Discussion

Our knowledge of chaetognath neurogenesis is mainly restricted to the description of the general morphology and organization of the nervous system using combinations of light microscopy, ultrastructural analyses, and generic immunohistochemical assays using antibodies directed against serotonin, FMRFamide-like peptides, RFamide-like peptides, acetylated or tyrosinated alpha tubulin [23–25, 43, 56, 60]. The molecular processes underlying neurogenesis are, however, still poorly understood for Chaetognatha due to the lack of data, resulting in difficulties when comparatively investigating nervous system development and evolution in bilaterians. This is the first comprehensive study that further explores the molecular processes involved in the nervous system development of *Spadella cephaloptera* hatchlings. Specifically, we focused on genes involved in NS formation that have been considered to play a role during mediolateral patterning of the bilaterian nervous system (e.g. [5, 9, 61]) to acquire insights into evolutionarily conserved features during NS development among chaetognaths and other bilaterians.

### Early patterns of neuronal development in young *Spadella* hatchlings

In the first 24 hours of postembryonic development, the central nervous system primarily comprises a dorsally located brain that is arranged as a circumoral loop and is linked to a VNC occupying most of the trunk (Fig. 1d, f) [25]. Subsequently, the brain develops an anterior and a posterior domain. The anterior domain gives rise to a second circumorally arranged brain component, including vestibular and esophageal ganglia [25, 43]. The posterior brain domain processes the input from sensory organs and may play a role in the regulation of motor behavior in response to sensory input changes. In adults, the anterior domain functions mainly in controlling the activity of several oral components related to feeding, including the grasping spines and musculature responsible for opening and closing the mouth [43].

To provide a better picture of the nervous system in 1 dph individuals of *S. cephaloptera*, we describe the spatial arrangement of post-mitotic differentiating neurons by studying *elav* expression [62]. While *elav* ^+^ cells are present in regions of the differentiating brain and VNC, our study highlights expression domains that have not been ascribed as neural regions before in chaetognaths [25, 60]. We detect strong *Sce-elav*-expression in a pair of cell clusters located posterior to the cerebral ganglion and laterally flanking the esophagus, which likely contribute to the nascent vestibular and esophageal ganglia. This paired structure is also *Sce-foxA* ^+^ and slightly *Sce-VAChT* ^+^. Furthermore, a similar expression domain is observed in the early juveniles where the vestibular ganglia are more defined. While the vestibular ganglia of 1 dph *S. cephaloptera* only become visible by 72 h post-hatching as revealed by synapsin and FMRFamide immunoreactivity [25], *Sce-elav* expression patterns in the head of the young hatchling suggest that the smaller and premature cephalic ganglia are already present and start developing at the same time as the cerebral ganglion.

### Conserved expression of *foxA* in the CNS and the developing foregut

*FoxA* is suggested to be involved in morphogenesis and differentiation during embryonic development [63, 64]. It is among those genes demonstrated to possess regulatory functions in the formation and development of the different digestive tract regions in a broad range of bilaterian lineages. *FoxA* is expressed in the foregut of the mollusks *Patella vulgata* [65] *and Crepidula fornicata* [66], in the ectodermal foregut and hindgut of annelids [67–70] and the planarians *Dugesia japonica* [71] and *Schmidtea polychroa* [72]. In addition, expression of *foxA* in the pharynx has also been reported for Phoronida [73], Ecdysozoa [74–77], and Chordata [78–80]. In *S. cephaloptera* hatchlings, *foxA-*expression is also observed in various parts of the digestive tract, particularly in cells lining the foregut, corroborating its conserved regulatory functions during bilaterian digestive tract formation and regionalization [81]. Moreover, *Sce-foxA-*expression is also detected in the developing brain and the VNC, which resembles the condition in annelids [67–69] and ecdysozoans [74, 82, 83]. While some annelids probably express *foxA* in the ventral neuroectoderm, *foxA* is expressed in the floor plate of the developing neural tube of chordates [80, 84–87]. This observation has been used as an argument in favor of a shared origin of the CNS among bilaterians ([6, 63], but see [16] for a different view). Although, at a later stage of neural development, it is interesting to note that *foxA* expression in the chaetognath hatchling is also observed in the ventral midline of the VNC. However, it remains to be investigated whether a similar expression pattern is also present during early ectoderm development of chaetognaths and whether this *foxA* domain is also conserved in other hitherto unstudied spiralians.

### Conserved expression of *nkx2.2* in the VNC

The VNC expression domain of *nkx2.2* is shared among protostomes, such as insects [61, 88], annelids [4, 10], platyhelminths [89], brachiopods, nemerteans, and rotifers [10]. In vertebrates, *nkx2.2* is also expressed in the developing neural tube. Hatchlings of *S. cephaloptera* also exhibit *nkx2.2* expression in the VNC, suggesting its conserved role during CNS development in chaetognaths and other bilaterians.

*Nkx2.2* is also expressed in cells lining the gut of young *Spadella* hatchlings, which is an expression domain that is also present in the developing embryonic and larval gut of the annelid *O. fusiformis* and early juvenile of the brachiopod *Terebratalia transversa* [10]. This indicates that *nkx2.2*, at least in Spiralia, might not only be involved in early neurogenesis, but also in gut differentiation. Nevertheless, more work is needed to demonstrate the functional role of *nkx2.2* during gut development.

#### Conserved expression of *pax6* in the CNS and eyes

The *pax6* expression pattern in *Spadella* hatchlings generally resembles the expression of the developing sensory organs and nervous systems in many bilaterians. Particularly, its role during eye development has been consistently demonstrated in many spiralians, including annelids [90, 91], cephalopods [92, 93], and nemerteans [94], and also in several species across major ecdysozoan and deuterostome lineages [95, 96]. The *Sce-pax6* homolog is also expressed in the developing eyes of hatchlings and weakens in later stages, suggesting its important regulatory roles during early post-embryonic eye development. Thus far, the only available data for *pax6* expression in the Gnathifera clade is from the rotifer *Epiphanes senta* [10]. The rotifer juveniles exhibit *pax6* expression as two bands at the lateral regions of the brain, which is an expression domain resembling that of *Spadella* hatchlings. However, it is unclear whether it is also detected in their cerebral eye. Other sensory structures in different protostomes also show *pax6* expression, such as the olfactory organ in squids [97, 98], palps and antennae in the annelid *P. dumerilii* [90], and the mushroom body of the fruit fly *D. melanogaster* [99]. Similarly, *Sce-pax6* was observed in different sensory organs of *Spadella* hatchlings such as the corona ciliata, implying that the functional role of *pax6* may have also expanded to the development of apomorphic morphological features of chaetognaths.

Moreover, various representatives of protostomes also express *pax6* in the cephalic ganglia and ventral nerve cords, including mollusks [97, 98, 100, 101], annelids [4, 91, 102], nemerteans [94], insects [103, 104], and crustaceans [105]. In the young *Spadella* hatchling, *pax6* is also expressed in the developing brain and the VNC, particularly as paired lateral domains in the brain and in the lateral somata clusters, respectively. The specific expression domains in the CNS indicate its functions in post-embryonic neuronal patterning, a role which has also been reported in different bilaterians [91, 103, 106]. Overall, our data on the *Sce-pax6* expression pattern in *Spadella* support the argument for the conserved expression of *pax6* in the developing eyes and CNS across bilaterian lineages.

#### *Pax3/7* may play a role in early neuronal patterning of the chaetognath VNC

The expression of *pax3/7* in the developing nervous system seems to be largely conserved in bilaterians. In several spiralians, *pax3/7* is consistently expressed in the CNS: In the annelids *P. dumerilii* [4] and *C. teleta* [107], *pax3/7* is expressed in two ventrolateral ectodermal bands. The brachiopod *T. transversa* also exhibits expression in the anterior ventral ectoderm of the larval trunk, and the cephalopod *Sepia officinalis* shows expression domains in ectodermal cells of different neural organs such as pedal ganglia and subesophageal region of the brain. Outside Spiralia, *pax3/7* is also shown to be expressed in neural regions during development. The *pax3/7* orthologs (gooseberry and gooseberry-neuro) in arthropods are also expressed in the neural ectoderm and are involved both in segmentation and the specification of neuroblast identity [108–112], while the chordate *pax3* and *pax7* are expressed in longitudinal bands in the developing neural tube of amphioxus, tunicates, and vertebrates [113–117]. In early-stage *Spadella* hatchlings, *pax3/7* is detected as two thin and bilateral stripes on the dorsal hemisphere of the VNC, which suggests that *pax3/7* also plays a role in the early neural patterning of the VNC, and therefore this role may be conserved in chaetognaths.

While *pax3* and *pax7* are generally specific markers for skeletal muscle developmental trajectories in vertebrates (e.g. [118, 119]), the involvement of *pax3/7* in mesoderm developmental processes is only known in few spiralians, such as the leech *Helobdella robusta* [120], and ecdysozoans such as *Pristionchus pacificus* [121]. Furthermore, the mesoderm during the late elongation stage of the brachiopod *N. anomala* also expresses *pax3/7* [10]. In this study, *Sce-pax3/7* is not detected in any mesodermal structures except in the presumably specialized peri-intestinal and lateral cells comprising the posterior septum [25]. Further work, however, will be necessary to understand how *pax3/7* functions in the formation and development of these mesodermal structures in *Spadella*.

#### Conserved expression of *msx* during muscle and nervous system development

*Msx* is known to play an important role in a variety of developmental processes in different bilaterians, more notably during neurogenesis and muscle formation. In Spiralia, *msx* expression is usually observed in the developing neural structures. The brachiopod *T. transversa* expresses *msx* in the ventral ectoderm, which includes the mantle and pedicle lobes [10]. Also, both annelids, *P. dumerilii* and *O. fusiformis* exhibit *msx* ^+^ cells in the ventral neuroectoderm, while the cerebral ganglia of the annelid *C. teleta* also show *msx* expression [122]. In the leech *H. robusta*, *msx* is expressed in all neurons of the anterior and posterior glial packets of the ventral nerve cord [123], and in the cephalopod *S. officinalis,* it is expressed in the developing branchial and stellate ganglia [92]. *Msx* is also expressed in two longitudinal bands in the neuroectoderm of *Drosophila* [124, 125], in the notochord and neural plate precursors of ascidians [126], and in the developing neuroectoderm during spinal cord development of vertebrates [125, 127]. The *Sce-msx* expression in *Spadella* hatchlings is largely observed in the VNC, but it is also detected in the brain of early juveniles. A strikingly intense level of expression is observed in some large neuronal cells in the lateral somata clusters of the young hatchling. It is, however, not clear whether these cells are identical to the aforementioned large *foxA* ^+^ neurons in the VNC of young hatchlings. In addition, *msx* is implicated during muscle differentiation and development of metazoans. The jellyfish *Podocoryne carnea* medusa expresses *msx* in the entocodon, a cell layer where the smooth and striated muscles emerge [128]. *Msh* (ortholog of *msx*) in *Drosophila* is expressed in muscle progenitors [124, 129] and in migratory limb muscle precursor cells in vertebrates [130]. Also, *N. anomala* exhibits *msx* expression in the mesoderm [10]. Muscle-specific expression in *Spadella* is also observed in the longitudinal muscles along the trunk of the young hatchling. In general, our data suggest that the role of *msx* during muscular and nervous system development seems to be conserved in chaetognaths.

#### Cholinergic domains in *Spadella* hatchlings

*ChAT* and *VAChT* have been used as specific markers to identify acetylcholine-containing (cholinergic) neurons. *ChAT* is expressed in the nerve cord and brain in the xenacoelomorphs *Meara stichopi* and *Isodiametra pulchra*, in a number of cells of the apical region of the embryo and larva, in the neuropil and lateral ventral nerve cord of the juvenile annelid *O. fusiformis*, and in the brain, the corona ciliata, and the mastax of the rotifer *E. senta* [10]. Structures positive for *ChAT* and *VAChT* are the brain and ventral nerve cord of the nemertean *L. ruber* and the anterior apical neuroectoderm of the brachiopod *T. transversa* [10]. In the annelid *P. dumerilii* and vertebrates, motor neurons that express *ChAT* and *VAChT* have been shown to emerge from the *pax6* ^+^/*nkx6* ^+^ progenitor domain of the ectoderm [4, 131, 132]. While this correspondence between cholinergic motor neuronal markers (i.e. *hb9*, *ChAT*, and *VAChT*) and *pax6* ^+^/*nkx6* ^+^ expression domains is evident in annelid and vertebrate model organisms, such expression colocalization is not observed in xenacoelomorphs and other spiralians [10]. In *Spadella* hatchlings, *ChAT* and *VAChT* are expressed in the VNC and in the brain of later stages. We have no data on *nkx6* expression, but both cholinergic neuronal markers are expressed within the *pax6* ^+^ domains. This expression colocalization is also observed in the cephalopod *S. officinalis*, where *pax6* expression is observed in the cholinergic-rich regions of the CNS [133]. However, the distribution of motor neurons in the *ChAT* ^+^ and *VAChT* ^+^ domains of the VNC remains to be identified.

### Expression of mediolateral patterning genes in the ventral nerve center of the *Spadella* hatchling

**Fig. 7:**
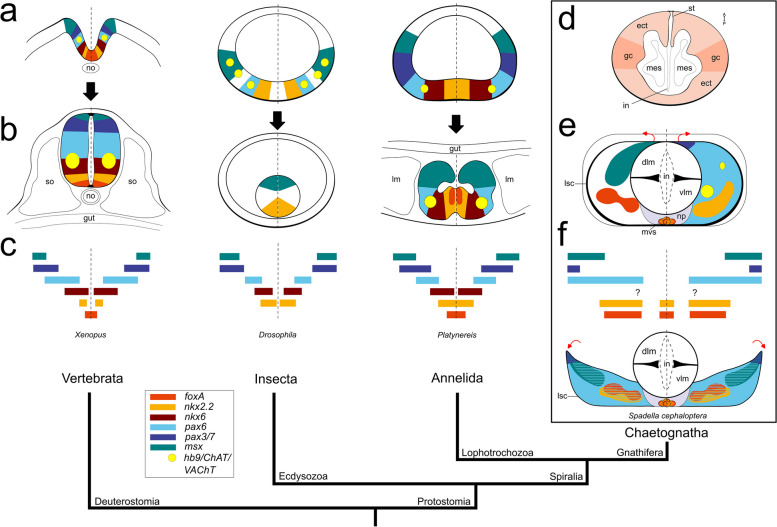
Comparison of the expression profiles of mediolateral patterning in the vertebrate, insect, annelid, and chaetognath trunk nervous system. Schematic drawings of regional patterning of mediolateral genes (**a**) during early stages of nerve cord development: neural plate folding in the frog *Xenopus* and ventral ectoderm development in the fruit fly *Drosophila* and the annelid *Platynereis*; and (**b**) during later ontogeny, when the folding of the neural tube of vertebrates proceeds and the ventral nerve cords of insects and annelids have further developed. The vertical dashed line indicates the neural midline. **c** The mediolateral extent of each neural patterning gene in the developing nerve cord is represented as a colored horizontal bar and the motor (*hb9*) and cholinergic (*ChAT* and *VAChT*) neuron subtype markers are labeled as yellow filled circles (modified from [5]). The vertebrate, insect, and annelid expression data are described in [61, 134–137], and [4, 138, 139], respectively. **d** A horizontal section of a 27 h post-laying embryo showing the location of the developing neuroblasts in the lateral side of the ectodermal layer that will eventually form the VNC (adapted from [42]). This is the earliest documented embryonic stage of *Spadella* where the VNC precursor cells (referred to as ganglion cells in [42]) were identified. **e** Expression patterns of mediolateral patterning genes schematized for the VNC of a *S. cephaloptera* hatchling (1 dph). **f** When the VNC is conceptually unfolded from the trunk longitudinal muscle into a flat nerve cord, the spatial extent of each gene conforms to a mediolateral system (mid and bottom images). Red arrows indicate the position and direction of the hypothetical unfolding process. Except for 7d, the dorsal and ventral side faces up and down, respectively. Question marks indicate that there is no information available. dlm: dorsal muscle; ect: ectoderm; gc: ganglion cells; in: intestine; lm: longitudinal muscle; lsc: lateral somata clusters; mes: mesodermal cells; mvs: medioventral somata clusters; no: notochord; np: neuropil; so: somites; st: stomodeum; vlm: ventral longitudinal muscle

Our study demonstrates that the genes *foxA, nkx2.2, pax6, pax3/7,* and *msx,* which have been proposed to be involved in the mediolateral patterning of the bilaterian nerve cords, are also expressed in the chaetognath ventral nerve center. Nevertheless, at first glance, no similarity in the mediolateral patterning sequence of the above-mentioned genes has been observed for hatchlings (Figs. 3, 4; Fig. 7e) or early juveniles of *S. cephaloptera* and the latter stage, the distinct gene expression patterning observed in hatchlings is even absent (Fig. 6). The lack of staggered expression patterns of the mediolateral patterning genes may be explained by the fact that no earlier embryonic stages have been studied for *S. cephaloptera* or that mediolateral patterning has been secondarily lost in chaetognaths [5, 12]. However, it could also be that mediolateral patterning is not evolutionarily conserved among bilaterians and has evolved convergently in different organisms [10].

The lateral somata clusters enclose four trunk muscle packages and the intestine. During subsequent ontogeny, the trunk muscle packages expand outward and separate from the intestine, resulting in a large coelomic space in between the two organs (Fig. 1i). While expanding, the lateral somata clusters break away from enveloping the trunk muscles, and eventually lie on the ventral side of adults with ventral longitudinal muscles and intestine dorsally (Fig. 1i). If the ventral nerve center of hatchlings were conceptually laterally unfolded from the longitudinal muscle into a flat structure (Fig. 7f), then the mediolateral sequence order of gene expression would be *foxA, nkx2.2, pax6, msx,* and *pax3/7*, resembling the condition observed in bilaterian models such as vertebrates [134, 135], *Drosophila* [61, 136, 137], and the annelid *P. dumerilii* [4, 138, 139] (Fig. 7a – c). Interestingly, a similar observation has been studied in the egg yolk-rich embryos of the cephalopod *S. officinalis* where mediolateral patterning is obscured by the unique mode of gastrulation of cephalopods [92].

## Conclusion

In this work, we provide insights on the molecular aspects of early post-embryonic neurogenesis in the benthic chaetognath *Spadella cephaloptera*. The *Sce-elav* expression reveals previously undescribed neural domains in *S. cephaloptera* hatchlings, including cell clusters located posterior to the cerebral ganglion and laterally flanking the esophagus, which likely contribute to the nascent vestibular and esophageal ganglia, and medioventral somata clusters of the VNC. Developmental genes and genes encoding transcription factors associated with neural differentiation during bilaterian nervous system development are also expressed in the neural domains of *S. cephaloptera* hatchlings, suggesting that their ancestral roles during bilaterian neurogenesis are likely conserved in Chaetognatha. Although these marker genes for neural patterning are expressed in specific neuronal cell subpopulations of the VNC, there is no evident staggered mediolateral patterning observed in the nerve cord of *Spadella* hatchlings, unless the latter is conceptually unfolded laterally from the longitudinal muscles into a flat structure. It would be necessary, however, to investigate earlier developmental stages directly after gastrulation to substantiate whether such mediolateral system is also present in chaetognaths.

### Supplementary Information

**Additional file 1: Table S1.** Genbank accession numbers of all sequences used in this study. Sequences that serve as outgroups are highlighted in yellow. The substitution model used in the phylogenetic analysis is also indicated.

**Additional file 2: Table S2.** Primer sequences and PCR annealing temperatures used for isolation of the *Spadella cephaloptera* gene sequences.

**Additional file 3: Figure S1–7. **Phylogenetic trees of genes of interest based on bilaterian protein sequences obtained from published literature and BLAST searches of the NCBI GenBank

**Additional file 4: Figure S8.** Expression patterns of *elav* in an early juvenile (7–10 dph) of *S. cephaloptera*. Gene transcripts are visualized with AP-Fast Blue (yellow) and cell nuclei with DAPI (purple). (a– d) Horizontal confocal sections of the head from (a) dorsal to (d) ventral. (d) An expression domain is detected lining the ventral esophagus, which is the location of the presumed sub-esophageal ganglion (arrowhead; also shown in panels *i* and *l*). (e—j) Transverse profiles of the head, from (e) anterior to (j) posterior. (h) Ellipses indicate the location of the eyes. Transverse section locations are marked in *a*. (k, l) Lateral profiles of the head along the (k) eye, exposing the lateral view of the vestibular ganglion and (l) longitudinal midline of the head. (m) Maximum projection of the entire trunk. (n) A medio-dorsal profile of (m) showing the longitudinal muscle cells and posterior terminus of the intestine. (o) Transverse profile of the trunk. (p) Lateral maximum projection of the trunk. (q) Lateral profile along the midline of trunk). (r) Maximum horizontal and (s) lateral projections of the tail. An expression domain (arrowhead) is detected in the anterior-most tail where the presumptive germ cells are developing (also shown in panels *n* and *t*) and in the lateral cells posterior to it (arrows). (t) Transverse profile of the anterior tail (refer to *r* for the section location). Scale bars: 50 μm, except panels (p) and (q) (100 μm). Asterisk indicates the position of the mouth opening. Orientation of specimens is indicated in the top right corner of each panel. cc, corona ciliata; cfo, ciliary fence organ; cg, cerebral ganglion; cp, cephalic adhesive papillae; cto, ciliary tuft organ; dlm, dorsal longitudinal muscle; es, esophagus; eg, esophageal ganglion; ey, eye; fn, fin; gmb, gut-muscle bundle; in, intestine; lsc, lateral somata clusters; mvs, medioventral somata clusters; tl, tail; vg, vestibular ganglion; vlm, ventral longitudinal muscle.

**Additional file 5: Figure S9.** Expression patterns of *foxA* in hatchlings (1 dph) and early juveniles (7–10 dph) of *S. cephaloptera*. Gene transcripts are visualized with AP-Fast Blue (yellow) and cell nuclei with DAPI (purple). (a – l) Confocal sections of a hatchling. (a) Horizontal maximum projection of the head. Eyes are encircled. (b) Mid head horizontal profile showing that *Sce-foxA* expression is prominent in cells arranged in a bulbous shape (red arrowheads, also visualized in c—g), cells lining the mouth and esophagus (solid outline), and in two cell clusters flanking the esophagus (dashed oval outline). (c) Ventral horizontal confocal section of the head. (d – f) Transverse profiles of the head, from (d) anterior to (f) posterior. (f) Eye locations are encircled. Transverse section locations are marked in *a*. (g, h) Lateral profiles of the head along the (g) eye and (h) midline of the head. (i, i') In the trunk, *Sce-foxA* expression domains are detected in the intestine (i, k; arrows), in lateral (i, i', j) and medioventral somata clusters (i', k), and in the ventroposterior trunk (l). (j) Higher magnification of the *Sce-foxA*^+^ large neuronal cells (left panel) and the DAPI channel with the expression boundary in dashed outline (right panel). (k) Lateral profile along the midline of gut-muscle bundle. (l) Transverse profile of the posterior trunk showing expression in ventral cells (arrowhead). Section location is indicated in *i’*. (m – w) Section profiles of an early juvenile. (m – p) Horizontal confocal sections of the head. Intense expression is observed in the (m) cerebral ganglion, (n) vestibular ganglia, and (o, p) cells around the mouth area. In *n* – *p*, the right panels only show the DAPI channel. (q) Transverse profiles of the anterior and (r) posterior cerebral ganglion. Vestibular ganglia are indicated in dashed outline. (s, t) Lateral profiles of the head. (s) Lateral projection or the whole head. (t) Lateral view along the longitudinal midline of the head. (u) Maximum projection, (v) lateral, and (w) transverse profile of the anterior tail showing expression in cells around the anus (arrowheads). Scale bars: 50 μm, except panels j (15 μm) and g, h, s, t, v (100 μm). The asterisk indicates the position of the mouth opening. Orientation of specimens is indicated in the top right corner of each panel. cc, corona ciliata; cfo, ciliary fence organ; cg, cerebral ganglion; cp, cephalic adhesive papillae; es, esophagus; fh, frontal horn; fn, fin; gmb, gut-muscle bundle; in, intestine; lms: longitudinal muscles somata; lp: lateral plate; lsc, lateral somata clusters; mvs, medioventral somata clusters; pep, perioral epidermis; vg, vestibular ganglion.

**Additional file 6: Figure S10.** Expression patterns of *Sce-nkx2.2* in *S. cephaloptera* hatchlings. Gene transcripts are visualized with AP-Fast Blue (yellow) and cell nuclei with DAPI (purple). (a) Horizontal maximum projection (ventral view) and (b) mid horizontal profile of the head. (c, d) Transverse profiles of the (c) anterior head and (d) along the future mouth (asterisk). (e, f) Lateral profiles of the (e) entire anterior half and along the midline exposing the expression in the (f) gut. (g) Horizontal maximum projection of the posterior trunk. (h) Ventral horizontal profile in the posterior trunk and (i) lateral profile along the trunk midline showing expression in mvs and cells in the ventroposterior lateral somata cluster (arrowhead). Scale bars: 50 μm. The asterisk indicates the position of the mouth opening. Orientation of specimens is in the top right corner of each panel. cg, cerebral ganglion; cp, cephalic adhesive papillae; es, esophagus; in, intestine; lsc, lateral somata clusters; mvs, medioventral somata clusters.

**Additional file 7: Figure S11.** Expression patterns of *Sce-pax6* in hatchlings (1 dph) and early juveniles (7–10 dph) of *S. cephaloptera*. Gene transcripts are visualized with AP-Fast Blue (yellow) and nuclei with DAPI (purple). (a – i) Section profiles of a hatchling. (a) Maximum horizontal projection of the anterior body. (b) Horizontal profile along the midplane of the anterior body. (c – e) Transverse profiles of the head. The location of the eyes is encircled and the corona ciliata is bordered by a dashed outline. (f) Lateral profile of the anterior body. Dashed outline indicates the anterior boundary of the lateral somata clusters. (g) Horizontal profile along the midplane of the trunk. (h) Horizontal maximum projection of the tail showing expression domains in sensory organs (arrowheads). (i) Transverse profile of the sensory organs on the lateral tail. Location of the section is indicated in *h*. (j – s) Section profiles of an early juvenile. (j) Maximum horizontal projection of the head. (k, l) Horizontal profiles along the (k) mid and (l) ventral plane. (*k, n*) The presumptive esophageal ganglia show a slight *Sce-pax6* expression (arrowheads). (m – o) Transverse profiles of the head. (p) Lateral profile along the midline of the anterior body. (q) Horizontal maximum projection of the half of the trunk and (q’) horizontal profile of the ventral plane of *q*. (r) Horizontal maximum projection of the tail with ciliary fence receptors in arrows. (s) Transverse profile along the ciliary fence organ of the tail (refer to *r* for the section location). Scale bars: 50 μm. The asterisk indicates the position of the mouth opening. Orientation of specimens is in the top right corner of each panel. cc, corona ciliata; co, ciliary organ; cg, cerebral ganglion; cp, cephalic adhesive papillae; es, esophagus; ey, eye; fh: frontal horn; fn, fin; gmb, gut-muscle bundle; in, intestine; lsc, lateral somata clusters; lms, longitudinal muscle somata; mvs, medioventral somata clusters; rco: retrocerebral organ.

**Additional file 8: Figure S12.** Expression patterns of *Sce-pax3/7* in hatchlings (1 dph) and early juveniles (7–10 dph) of *S. cephaloptera*. Gene transcripts are visualized with AP-Fast Blue (yellow) and cell nuclei with DAPI (purple). (a – h) Section profiles of a hatchling. (a) Horizontal maximum projection of the head. (b, b’) Higher magnification of the two *Sce-pax3/7*^+^ cells in the anterior terminal of lateral somata clusters (left panels) and the DAPI channel with the expression boundary in dashed outline (right panels). (c – e) Horizontal confocal sections of the trunk-tail boundary. (d) Magnified view of the horizontal midline showing expression in specialized mesodermal cells (presumptive peri-intestinal cells in arrow and lateral cells in arrowheads) separating the germ cells (dashed outline). (e) Expression domain in the ventral cells (red arrowhead) posterior to the medioventral somata clusters. (f, g) Lateral profile of the trunk along the (f) lateral somata clusters exhibiting the longitudinal expression pattern and (g) longitudinal midline (gut-muscle bundle) showing the *Sce-pax3/7*^+^ cells in the posterior terminal of the gut. (h) Transverse profile of the posterior trunk (section location in *c*). Encircled is the gut-muscle bundle. (i – p) Section profiles of a juvenile. (i, j) Horizontal profiles of the head showing expression in the cephalic ganglia. (k) Lateral view of the whole head. (l) Horizontal maximum projection of the tail. (m, n) Horizontal profiles of the trunk-tail boundary showing the expression domains in (m) posterior septum in mid-section and (n) anus in the ventral plane. (o) Lateral profile along the tail longitudinal midline. (p) Transverse profile along the posterior septum and anal region. Scale bars: 50 μm, except panels b, b’, d (15 μm). The asterisk indicates the position of the mouth opening. Orientation of specimens is in the top right corner of each panel. an, anus; cc, corona ciliata; cfo, ciliary fence organ; cg, cerebral ganglion; es, esophagus; eg: esophageal ganglion; gmb, gut-muscle bundle; lsc, lateral somata clusters; lms, longitudinal muscle cells; mvs, medioventral somata clusters; posterior septum; tl, tail; vg, vestibular ganglion.

**Additional file 9: Figure S13.** Expression patterns of *msx* in hatchlings (1 dph) and juveniles (7–10 dph) of *S. cephaloptera*. Gene transcripts are visualized with AP-Fast Blue (yellow) and cell nuclei with DAPI (purple). (a – g) Section profiles of a hatchling. (a) Horizontal maximum projection of the head. Eye is encircled. (b, b’) Higher magnification of the two *Sce-msx*^+^ cells in the head (left panels) and the DAPI channel with the expression boundary in dashed outline (right panels). (c) Cells in b and b’ (arrowheads) in transverse view. (d) Transverse profile showing expression in cells in the anteroventral corona ciliata (arrows). (e) Horizontal maximum projection of the trunk. (f, f’) Horizontal profiles of the midtrunk showing *Sce-msx* signal in large neuronal cells of the lateral somata clusters and in muscle fibers (arrowheads). *f* is a dorsal section, while f’ is along the midplane of the intestine. (g) Higher magnification of the *Sce-msx*^+^ large neuronal cells (left panel) and the DAPI channel with the expression boundary in dashed outline (right panel). (h – j) Section profiles of a juvenile. (h, i) Horizontal profiles of the head. Expression is more prominent in the (h) posterior cerebral ganglion and in the (i) vestibular and esophageal ganglia. (j) Maximum horizontal projection of the trunk and (j’) horizontal profile of the mid trunk. Scale bars: 50 μm, except panels b, b’, and g (15 μm). Asterisk indicates the position of the mouth opening. Orientation of specimens is indicated in the top right corner of each panel. cc, corona ciliata; cg, cerebral ganglion; es, esophagus; eg, esophageal ganglion; in, intestine; lsc, lateral somata clusters; mvs: medioventral somata clusters; tl: tail; vg, vestibular ganglion.

**Additional file 10: Figure S14.** Expression patterns of *Sce-ChAT* in hatchlings (1 dph) and early juveniles (7–10 dph) of *S. cephaloptera*. Gene transcripts are visualized with AP-Fast Blue (yellow) and cell nuclei with DAPI (purple). (a-c) Section profiles of a hatchling. (a) Maximum horizontal projection of the anterior half of the body. (b) Horizontal profile of the trunk showing *Sce-ChAT* expression in lateral cell somata adjacent to the muscle cells (arrowheads). (c) Lateral profile of anterior half of the body. (d) Late-stage encapsulated embryo with signal (arrowheads) in medial lateral somata cluster (dashed outline). The egg capsule around the embryo is strongly stained. (e – o) Section profiles of a juvenile. (e) Maximum horizontal projection of the head. (f – h) Horizontal profiles of the head from dorsal (f) to ventral (h). (g) Expression pattern is shown on the left panel and DAPI-only channel on the right panel for a better representation of the expression extent. (i – l) Transverse profiles of the head from (i) anterior to (l) posterior. In (k), the location of the eyes is encircled. (m) Lateral profiles of the whole anterior half. (n) Lateral profile along the longitudinal midline of the anterior half. (o) Horizontal maximum projection of the posterior trunk and anterior tail. Scale bars: 50 μm. Asterisk indicates the position of the mouth opening. Orientation of specimens is indicated in the top right corner of each panel. cc, corona ciliata; cg, cerebral ganglion; es, esophagus; eg, esophageal ganglion; ey, eye; in, intestine; lsc, lateral somata clusters; tl, tail; vg, vestibular ganglion.

**Additional file 11: Figure S15.** Expression patterns of *Sce-VAChT* in hatchlings (1 dph) and early juveniles (7–10 dph) of *S. cephaloptera*. Gene transcripts are visualized with AP-Fast Blue (yellow) and cell nuclei with DAPI (purple). (a – d, f, f’) Section profiles of a hatchling. (a) Maximum horizontal projection of the anterior half of the animal. (b) Horizontal profile of the trunk showing *Sce-VAChT* expression in lateral cell somata adjacent to the muscle cells (arrowheads). (c) Transverse profile of the head showing expression domains in cells flanking the oral cavity (dorsal to the asterisk). (d) Lateral profile of the anterior half. (e, g, g’) Section profiles of a juvenile. (e) Horizontal maximum projection of the trunk. (f, f’, g, g’) Higher magnification of the *Sce-VAChT*^+^ large neuronal cells (left panels) and the DAPI channel with the expression boundary in dashed outline (right panels) in hatchling and early juveniles, respectively. Scale bars: 50 μm, except panels f, f’, g, and g’ (15 μm). cc, corona ciliata; cg, cerebral ganglion; in, intestine; mvs, medioventral somata clusters; lsc, lateral somata clusters.

## Data Availability

All sequence data are available on Genbank. For the purpose of open access, the author has applied a CC BY public copyright license to any Author Accepted Manuscript version arising from this submission.

## Abbreviations

AIC: Akaike Information Criterion
CNS: Central Nervous System
dph: Days post hatch
VNC: Ventral nerve center
PCR: Polymerase Chain Reaction
DIG: Digoxigenin
WM-FISH: Whole-Mount Fluorescent In-Situ Hybridization
PBS: Phosphate Buffer Saline
NAMP: Naphthol-AS-MX-phosphate
DAPI: 4′,6-Diamidino-2-phenylindole

## References

[1] Schmidt-Rhaesa A. The evolution of organ systems. Oxford: Oxford University Press; 2007. p. 1–396.

[2] Ax P (1996). Multicellular animals: a new approach to the phylogenetic order in nature.

[3] Wanninger A. Evolutionary developmental biology of invertebrates 6: Deuterostomia. Evol Dev Biol Invertebr 6 Deuterostomia. 2015;6:1–214.

[4] Denes AS, Jékely G, Steinmetz PRH, Raible F, Snyman H, Prud’homme B (2007). Molecular Architecture of Annelid Nerve Cord Supports Common Origin of Nervous System Centralization in Bilateria. Cell..

[5] Arendt D. Animal evolution: convergent nerve cords? Curr Biol. 2018;28:R225–7. 10.1016/j.cub.2018.01.056 29510113

[6] Arendt D, Denes AS, Jékely G, Tessmar-Raible K. The evolution of nervous system centralization. Philos Trans R Soc B Biol Sci. 2008. 1523–8. 10.1098/rstb.2007.2242 PMC2614231 18192182

[7] Arendt D, Tosches MA, Marlow H. From nerve net to nerve ring, nerve cord and brain-evolution of the nervous system. Nat Rev Neurosci. 2016. 61–72. 10.1038/nrn.2015.15 26675821

[8] Moroz LL. On the independent origins of complex brains and neurons. Brain Behav Evol. 2009;74:177–90. 10.1159/000258665 PMC2855278 20029182

[9] Holland LZ, Carvalho JE, Escriva H, Laudet V, Schubert M, Shimeld SM, et al. Evolution of bilaterian central nervous systems: A single origin? Evodevo. 2013;4:1–27. 10.1186/2041-9139-4-27 PMC3856589 24098981

[10] Martín-Durán JM, Pang K, Børve A, Lê HS, Furu A, Cannon JT (2018). Convergent evolution of bilaterian nerve cords. Nature.

[11] Chisholm AD, Horvitz HR (1995). Patterning of the caenorhabditis elegans head region by the pax-6 family member vab-3. Nature.

[12] Treffkorn S, Kahnke L, Hering L, Mayer G. Expression of NK cluster genes in the onychophoran Euperipatoides rowelli: Implications for the evolution of NK family genes in nephrozoans. Evodevo. 2018;9:1–32. 10.1186/s13227-018-0105-2 PMC6050708 30026904

[13] Franke FA, Schumann I, Hering L, Mayer G (2015). Phylogenetic analysis and expression patterns of Pax genes in the onychophoran Euperipatoides rowelli reveal a novel bilaterian Pax subfamily. Evol Dev.

[14] Eriksson BJ, Samadi L, Schmid A (2013). The expression pattern of the genes engrailed, pax6, otd and six3 with special respect to head and eye development in Euperipatoides kanangrensis Reid 1996 (Onychophora: Peripatopsidae). Dev Genes Evol.

[15] Kaul-Strehlow S, Urata M, Praher D, Wanninger A. Neuronal patterning of the tubular collar cord is highly conserved among enteropneusts but dissimilar to the chordate neural tube. Sci Rep. 2017;7:1–10. 10.1038/s41598-017-07052-8 PMC5539250 28765531

[16] Lowe CJ, Terasaki M, Wu M, Freeman RM, Runft L, Kwan K (2006). Dorsoventral patterning in hemichordates: Insights into early chordate evolution. PLoS Biol.

[17] Lowe CJ, Wu M, Salic A, Evans L, Lander E, Stange-Thomann N (2003). Anteroposterior patterning in hemichordates and the origins of the chordate nervous system. Cell.

[18] Martin-Duran JM, Hejnol A. A developmental perspective on the evolution of the nervous system. Dev Biol. 2021;475:181–92. 10.1016/j.ydbio.2019.10.003 31610146

[19] Kaul-Strehlow S, Rottinger E. Hemichordata. Evol Dev Biol Invertebr 6 Deuterostomia. 2015.6:59–90.

[20] Hartenstein V, Stollewerk A. The evolution of early neurogenesis. Dev Cell. 2015;32(4):390–407. 10.1016/j.devcel.2015.02.004 PMC5987553 25710527

[21] Kapp H, Westheide W, Rieger G (2007). Chaetognatha – Pfeilwürmer. Spez Zool.

[22] Telford MJ. Affinity for arrow worms. Nature. 2004;431:254–6. 10.1038/431254b 15372015

[23] Harzsch S, Muller CHG. A new look at the ventral nerve centre of Sagitta: Implications for the phylogenetic position of Chaetognatha (arrow worms) and the evolution of the bilaterian nervous system. Front Zool. 2007;4:1–14. 10.1186/1742-9994-4-14 PMC1885248 17511857

[24] Harzsch S, Wanninger A (2010). Evolution of invertebrate nervous systems: The Chaetognatha as a case study. Acta Zool.

[25] Rieger V, Perez Y, Müller CHG, Lacalli T, Hansson BS, Harzsch S (2011). Development of the nervous system in hatchlings of Spadella cephaloptera (Chaetognatha), and implications for nervous system evolution in Bilateria. Dev Growth Differ.

[26] Marletaz F, Peijnenburg KTCA, Goto T, Satoh N, Rokhsar DS. A new spiralian phylogeny places the enigmatic arrow worms among Gnathiferans. Curr Biol. 2019;29:312–318.e3. 10.1016/j.cub.2018.11.042 30639106

[27] Zrzavý J, Mihulka S, Kepka P, Bezděk A, Tietz D (1998). Phylogeny of the metazoa based on morphological and 18s ribosomal DNA evidence. Cladistics.

[28] Halanych KM (1996). Testing hypotheses of chaetognath origins: Long branches revealed by 18S ribosomal DNA. Syst Biol.

[29] Peterson KJ, Eernisse DJ (2001). Animal phylogeny and the ancestry of bilaterians: Inferences from morphology and 18S rDNA gene sequences. Evol Dev.

[30] Paps J, Baguñà J, Riutort M (2009). Lophotrochozoa internal phylogeny: New insights from an up-to-date analysis of nuclear ribosomal genes. Proc R Soc B Biol Sci.

[31] Mallatt J, Winchell CJ (2002). Testing the new animal phylogeny: First use of combined large-subunit and small-subunit rRNA gene sequences to classify the protostomes. Mol Biol Evol.

[32] Littlewood DTJ, Telford MJ, Clough KA, Rohde K (1998). Gnathostomulida - an enigmatic metazoan phylum from both morphological and molecular perspectives. Mol Phylogenet Evol.

[33] Matus DQ, Copley RR, Dunn CW, Hejnol A, Eccleston H, Halanych KM, et al. Broad taxon and gene sampling indicate that chaetognaths are protostomes. Curr Biol. 2006;16:R575–76. 10.1016/j.cub.2006.07.017 16890509

[34] Papillon D, Perez Y, Caubit X, Le Parco Y (2004). Identification of Chaetognaths as protostomes is supported by the analysis of their mitochondrial genome. Mol Biol Evol.

[35] Dunn CW, Hejnol A, Matus DQ, Pang K, Browne WE, Smith SA (2008). Broad phylogenomic sampling improves resolution of the animal tree of life. Nature.

[36] Philippe H, Brinkmann H, Copley RR, Moroz LL, Nakano H, Poustka AJ (2011). Acoelomorph flatworms are deuterostomes related to Xenoturbella. Nature.

[37] Giribet G, Distel DL, Polz M, Sterrer W, Wheeler WC (2000). Triploblastic relationships with emphasis on the Acoelomates and the position of Gnathostomulida, Cycliophora, Plathelminthes, and Chaetognatha: A combined approach of 18S rDNA sequences and morphology. Syst Biol.

[38] Helfenbein KG, Fourcade HM, Vanjani RG, Boore JL (2004). The mitochondrial genome of Paraspadella gotoi is highly reduced and reveals that chaetognaths are a sister group to protostomes. Proc Natl Acad Sci U S A.

[39] Marletaz F, Martin E, Perez Y, Papillon D, Caubit X, Lowe CJ, et al. Chaetognath phylogenomics: a protostome with deuterostome-like development. Curr Biol. 2006;16:R577–78. 10.1016/j.cub.2006.07.016 16890510

[40] Telford MJ, Holland PWH (1993). The phylogenetic affinities of the chaetognaths: A molecular analysis. Mol Biol Evol.

[41] Papillon D, Perez Y, Fasano L, Le Parco Y, Caubit X. Hox gene survey in the chaetognath Spadella cephaloptera: Evolutionary implications. Dev Genes Evol. 2003;213:143–8. 10.1007/s00427-003-0306-z 12690453

[42] John CC (1933). Memoirs: habits, structure, and development of Spadella cephaloptera. J Cell Sci..

[43] Rieger V, Perez Y, Müller CHG, Lipke E, Sombke A, Hansson BS (2010). Immunohistochemical analysis and 3D reconstruction of the cephalic nervous system in Chaetognatha: Insights into the evolution of an early bilaterian brain?. Invertebr Biol.

[44] Bone Q, Pulsford A (1984). The Sense Organs and Ventral Ganglion of Sagitta (Chaetognatha). Acta Zool.

[45] Goto T, Yoshida M. Photoreception in Chaetognatha. In: Photoreception and vision in invertebrates. NATO ASI series. Springer. 1984;74:727–42.

[46] Chaetognatha SG, Harrison F, Ruppert E (1997). Microsc Anat Invertebr Hemichordata, Chaetognatha, Invertebr Chordates.

[47] Wollesen T, Rodriguez Monje S V, Oel AP, Arendt D. Characterization of eyes, photoreceptors, and opsins in developmental stages of the arrow worm Spadella cephaloptera (Chaetognatha). J Exp Zool Part B Mol Dev Evol. 2023. [cited 2023 Mar 31]; Available from: https://onlinelibrary.wiley.com/doi/10.1002/jez.b.23193 10.1002/jez.b.23193 PMC10952353 36855226

[48] Altschul SF, Gish W, Miller W, Myers EW, Lipman DJ (1990). Basic local alignment search tool. J Mol Biol..

[49] Thompson JD, Gibson TJ, Higgins DG. Multiple sequence alignment using ClustalW and ClustalX. Curr Protoc Bioinforma. 2003;00:2.3.1–2.3.22. 10.1002/0471250953.bi0203s00 18792934

[50] Capella-Gutiérrez S, Silla-Martínez JM, Gabaldón T (2009). trimAl: A tool for automated alignment trimming in large-scale phylogenetic analyses. Bioinformatics.

[51] Darriba D, Taboada GL, Doallo R, Posada D (2011). ProtTest 3: Fast selection of best-fit models of protein evolution. Bioinformatics.

[52] Ronquist F, Huelsenbeck JP (2003). MrBayes 3: Bayesian phylogenetic inference under mixed models. Bioinformatics.

[53] Lauter G, Soll I, Hauptmann G. Two-color fluorescent in situ hybridization in the embryonic zebrafish brain using differential detection systems. BMC Dev Biol. 2011;11:1–43. 10.1186/1471-213X-11-43 PMC3141750 21726453

[54] Schindelin J, Arganda-Carreras I, Frise E, Kaynig V, Longair M, Pietzsch T, et al. Fiji: An open-source platform for biological-image analysis. Nat Methods. 2012;9:676–82. 10.1038/nmeth.2019 PMC3855844 22743772

[55] Harzsch S, Müller CHG, Rieger V, Perez Y, Sintoni S, Sardet C (2009). Fine structure of the ventral nerve centre and interspecific identification of individual neurons in the enigmatic Chaetognatha. Zoomorphology.

[56] Goto T, Katayama-Kumoi Y, Tohyama M, Yoshida M (1992). Distribution and development of the serotonin-and RFamide-like immunoreactive neurons in the arrowworm, Paraspadella gotoi (Chaetognatha). Cell Tissue Res.

[57] Goto T, Yoshida M. Nervous system in Chaetognatha. Nerv Syst Invertebr. Boston: Springer US; 1987. p. 461–81.

[58] Li L, Vaessin H (2000). Pan-neural prospero terminates cell proliferation during Drosophila neurogenesis. Genes Dev.

[59] Pascale A, Amadio M, Quattrone A. Defining a neuron: Neuronal ELAV proteins. Cell Mol Life Sci. 2008;65:128–40. 10.1007/s00018-007-7017-y PMC11131659 17928954

[60] Perez Y, Rieger V, Martin E, Muller CHG, Harzsch S. Neurogenesis in an early protostome relative: progenitor cells in the ventral nerve center of chaetognath hatchlings are arranged in a highly organized geometrical pattern. J Exp Zool Part B Mol Dev Evol. 2013;320:179–93. 10.1002/jez.b.22493 23483730

[61] Arendt D, Nubler-Jung K. Comparison of early nerve cord development in insects and vertebrates. Development. 1999;126(11):2309–25. 10.1242/dev.126.11.2309 10225991

[62] Colombrita C, Silani V, Ratti A. ELAV proteins along evolution: Back to the nucleus? Mol Cell Neurosci. 2013;56:447–55. 10.1016/j.mcn.2013.02.003 23439364

[63] Kaestner KH. The FoxA factors in organogenesis and differentiation. Curr Opin Genet Dev. 2010;20(5):527–32. 10.1016/j.gde.2010.06.005 PMC2943037 20591647

[64] Carlsson P, Mahlapuu M. Forkhead transcription factors: Key players in development and metabolism. Dev Biol. 2002;250(1):1–23. 10.1006/dbio.2002.0780 12297093

[65] Lartilot N, Le Gouar M, Adoutte A (2002). Expression pattern of fork head and goosecoid homologues in the mollusc Patella vulgata supports the ancestry of the anterior mesendoderm across Bilateria. Dev Genes Evol.

[66] Perry KJ, Lyons DC, Truchado-Garcia M, Fischer AHL, Helfrich LW, Johansson KB (2015). Deployment of regulatory genes during gastrulation and germ layer specification in a model spiralian mollusc Crepidula. Dev Dyn.

[67] Boyle MJ, Seaver EC (2008). Developmental expression of foxA and gata genes during gut formation in the polychaete annelid. Capitella sp I Evol Dev.

[68] Boyle MJ, Seaver EC. Expression of FoxA and GATA transcription factors correlates with regionalized gut development in two lophotrochozoan marine worms: Chaetopterus (Annelida) and Themiste lageniformis (Sipuncula). Evodevo. 2010;1:1–18. 10.1186/2041-9139-1-2 PMC2938726 20849645

[69] Kostyuchenko RP, Kozin VV, Filippova NA, Sorokina EV (2019). FoxA expression pattern in two polychaete species, Alitta virens and Platynereis dumerilii: Examination of the conserved key regulator of the gut development from cleavage through larval life, postlarval growth, and regeneration. Dev Dyn.

[70] Arenas-Mena C. Embryonic expression of HeFoxA1 and HeFoxA2 in an indirectly developing polychaete. Dev Genes Evol. 2006;216:727–36. 10.1007/s00427-006-0099-y 17031669

[71] Koinuma S, Umesono Y, Watanabe K, Agata K (2000). Planaria FoxA (HNF3) homologue is specifically expressed in the pharynx-forming cells. Gene.

[72] Martín-Durán JM, Amaya E, Romero R (2010). Germ layer specification and axial patterning in the embryonic development of the freshwater planarian Schmidtea polychroa. Dev Biol.

[73] Andrikou C, Passamaneck YJ, Lowe CJ, Martindale MQ, Hejnol A. Molecular patterning during the development of Phoronopsis harmeri reveals similarities to rhynchonelliform brachiopods. Evodevo. 2019;10:1–15. 10.1186/s13227-019-0146-1 PMC6907167 31867094

[74] Janssen R, Schomburg C, Prpic NM, Budd GE. A comprehensive study of arthropod and onychophoran Fox gene expression patterns. PLoS One. 2022;17:1–42. 10.1371/journal.pone.0270790 PMC9269926 35802758

[75] Lee HH, Frasch M (2004). Survey of forkhead domain encoding genes in the drosophila genome: classification and embryonic expression patterns. Dev Dyn.

[76] Janssen R, Budd GE (2017). Investigation of endoderm marker-genes during gastrulation and gut-development in the velvet worm Euperipatoides kanangrensis. Dev Biol.

[77] Martín-Durán JM, Janssen R, Wennberg S, Budd GE, Hejnol A (2012). Deuterostomic development in the protostome Priapulus caudatus. Curr Biol.

[78] Taguchi S, Tagawa K, Humphreys T, Nishino A, Satoh N, Harada Y (2000). Characterization of a hemichordate fork head/HNF-3 gene expression. Dev Genes Evol.

[79] Oliveri P, Walton KD, Davidson EH, McClay DR (2006). Repression of mesodermal fate by foxa, a key endoderm regulator of the sea urchin embryo. Development.

[80] Kaufmann E, Knöchel W (1996). Five years on the wings of fork head. Mech Dev.

[81] Technau U, Scholz CB. Origin and evolution of endoderm and mesoderm. Int J Dev Biol. 2003;47(4-8):531–9. 14756329

[82] Weigel D, Jürgens G, Küttner F, Seifert E, Jäckle H (1989). The homeotic gene fork head encodes a nuclear protein and is expressed in the terminal regions of the Drosophila embryo. Cell.

[83] Kalb JM, Lau KK, Goszczynski B, Fukushige T, Moons D, Okkema PG, et al. Pha-4 is Ce-fkh-1, a fork head/HNF-3α,β,γ homolog that functions in organogenesis of the C. Elegans pharynx. Development. 1998;125:1–10. 10.1242/dev.125.12.2171 9584117

[84] Corbo JC, Erives A, Di Gregorio A, Chang A, Levine M (1997). Dorsoventral patterning of the vertebrate neural tube is conserved in a protochordate. Development.

[85] Olsen CL, Jeffery WR (1997). A forkhead gene related to HNF-3β is required for gastrulation and axis formation in the ascidian embryo. Development.

[86] Shimeld SM (1997). Characterisation of amphioxus HNF-3 genes: Conserved expression in the notochord and floor plate. Dev Biol.

[87] Sasaki H, Hogan BLM (1994). HNF-3β as a regulator of floor plate development. Cell.

[88] Weiss JB, Von Ohlen T, Mellerick DM, Dressler G, Doe CQ, Scott MP (1998). Dorsoventral patterning in the Drosophila central nervous system: The intermediate neuroblasts defective homeobox gene specifies intermediate column identity. Genes Dev.

[89] Scimone ML, Kravarik KM, Lapan SW, Reddien PW (2014). Neoblast specialization in regeneration of the planarian schmidtea mediterranea. Stem Cell Reports.

[90] Arendt D, Tessmar K, Medeiros de Campos-Baptista MI, Dorresteijn A, Wittbrodt J (2002). Development of pigment-cup eyes in the polychaete Platynereis dumerilii and evolutionary conservation of larval eyes in bilateria. Development..

[91] Klann M, Seaver EC (2019). Functional role of pax6 during eye and nervous system development in the annelid Capitella teleta. Dev Biol.

[92] Buresi A, Andouche A, Navet S, Bassaglia Y, Bonnaud-Ponticelli L, Baratte S (2016). Nervous system development in cephalopods: How egg yolk-richness modifies the topology of the mediolateral patterning system. Dev Biol.

[93] Navet S, Andouche A, Baratte S, Bonnaud L (2009). Shh and Pax6 have unconventional expression patterns in embryonic morphogenesis in Sepia officinalis (Cephalopoda). Gene Expr Patterns.

[94] Loosli F, Kmita-Cunisse M, Gehring WJ (1996). Isolation of a Pax-6 homolog from the ribbonworm Lineus sanguineus. Proc Natl Acad Sci U S A.

[95] Kozmik Z. Pax genes in eye development and evolution. Curr Opin Genet Dev. 2005;15(4):430–8. 10.1016/j.gde.2005.05.001 15950457

[96] Quiring R, Walldorf U, Kloter U, Gehring WJ (1994). Homology of the eyeless gene of drosophila to the small eye gene in mice and aniridia in humans. Science (80-).

[97] Tomarev SI, Callaerts P, Kos L, Zinovieva R, Halder G, Gehring W (1997). Squid Pax-6 and eye development. Proc Natl Acad Sci U S A.

[98] Hartmann B, Lee PN, Kang YY, Tomarev S, De Couet HG, Callaerts P (2003). Pax6 in the sepiolid squid Euprymna scolopes: Evidence for a role in eye, sensory organ and brain development. Mech Dev.

[99] Kurusu M, Nagao T, Walldorf U, Flister S, Gehring WJ, Furukubo-Tokunaga K (2000). Genetic control of development of the mushroom bodies, the associative learning centers in the Drosophila brain, by the eyeless, twin of eyeless, and dachshund genes. Proc Natl Acad Sci U S A.

[100] Vocking O, Kourtesis I, Hausen H. Posterior eyespots in larval chitons have a molecular identity similar to anterior cerebral eyes in other bilaterians. Evodevo. 2015;6:1–14. 10.1186/s13227-015-0036-0 PMC4689004 26702352

[101] Scherholz M, Redl E, Wollesen T, De Oliveira AL, Todt C, Wanninger A. Ancestral and novel roles of Pax family genes in mollusks. BMC Evol Biol. 2017;17:1–20. 10.1186/s12862-017-0919-x PMC5356317 28302062

[102] Quigley IK, Xie X, Shankland M (2007). Hau-Pax6A expression in the central nervous system of the leech embryo. Dev Genes Evol.

[103] Kammermeier L, Leemans R, Hirth F, Flister S, Wenger U, Walldorf U (2001). Differential expression and function of the Drosophila Pax6 genes eyeless and twin of eyeless in embryonic central nervous system development. Mech Dev.

[104] Yang X, Weber M, ZarinKamar N, Posnien N, Friedrich F, Wigand B (2009). Probing the Drosophila retinal determination gene network in Tribolium (II): The Pax6 genes eyeless and twin of eyeless. Dev Biol.

[105] Blackburn DC, Conley KW, Plachetzki DC, Kempler K, Battelle BA, Brown NL (2008). Isolation and expression of Pax6 and atonal homologues in the American horseshoe crab, Limulus polyphemus. Dev Dyn.

[106] Dulcis D, Lippi G, Stark CJ, Do LH, Berg DK, Spitzer NC (2017). Neurotransmitter Switching Regulated by miRNAs Controls Changes in Social Preference. Neuron.

[107] Seaver EC, Yamaguchi E, Richards GS, Meyer NP. Expression of the pairrule gene homologs runt, Pax3/7, even-skipped-1 and even-skipped-2 during larval and juvenile development of the polychaete annelid Capitella teleta does not support a role in segmentation. Evodevo. 2012;3:1–18. 10.1186/2041-9139-3-8 PMC3359188 22510249

[108] Skeath JB, Zhang Y, Holmgren R, Carroll SB, Doe CQ (1995). Specification of neuroblast identity in the drosophila embryonic central nervous system by gooseberry-distal. Nature.

[109] Buenzow DE, Holmgren R (1995). Expression of the Drosophila gooseberry Locus Defines a Subset of Neuroblast Lineages in the Central Nervous System. Dev Biol.

[110] He H, Noll M (2013). Differential and redundant functions of gooseberry and gooseberry neuro in the central nervous system and segmentation of the Drosophila embryo. Dev Biol.

[111] Gutjahr T, Patel NH, Li X, Goodman CS, Noll M (1993). Analysis of the gooseberry locus in Drosophila embryos: Gooseberry determines the cuticular pattern and activates gooseberry neuro. Development.

[112] Zhang Y, Ungar A, Fresquez C, Holmgren R (1994). Ectopic expression of either the Drosophila gooseberry-distal or proximal gene causes alterations of cell fate in the epidermis and central nervous system. Development.

[113] Goulding MD, Chalepakis G, Deutsch U, Erselius JR, Gruss P (1991). Pax-3, a novel murine DNA binding protein expressed during early neurogenesis. EMBO J.

[114] Jostes B, Walther C, Gruss P (1990). The murine paired box gene, Pax7, is expressed specifically during the development of the nervous and muscular system. Mech Dev.

[115] Basch ML, Bronner-Fraser M, García-Castro MI (2006). Specification of the neural crest occurs during gastrulation and requires Pax7. Nature.

[116] Kim K, Orvis J, Stolfi A. Pax3/7 regulates neural tube closure and patterning in a non-vertebrate chordate. Front Cell Dev Biol. 2022;10:1–10. 10.3389/fcell.2022.999511 PMC9511217 36172287

[117] Monsoro-Burq AH. PAX transcription factors in neural crest development. Semin Cell Dev Biol. 2015;44:87–96. 10.1016/j.semcdb.2015.09.015 26410165

[118] Relaix F, Rocancourt D, Mansouri A, Buckingham M (2005). A Pax3/Pax7-dependent population of skeletal muscle progenitor cells. Nature.

[119] Kassar-Duchossoy L, Giacone E, Gayraud-Morel B, Jory A, Gomès D, Tajbakhsh S (2005). Pax3/Pax7 mark a novel population of primitive myogenic cells during development. Genes Dev.

[120] Woodruff JB, Mitchell BJ, Shankland M (2007). Hau-Pax3/7A is an early marker of leech mesoderm involved in segmental morphogenesis, nephridial development, and body cavity formation. Dev Biol.

[121] Yi B, Bumbarger D, Sommer RJ (2009). Genetic evidence for pax-3 function in myogenesis in the nematode Pristionchus pacificus. Evol Dev.

[122] Schuster HC, Hirth F (2023). Phylogenetic tracing of midbrain-specific regulatory sequences suggests single origin of eubilaterian brains. Sci Adv..

[123] Master VA, Kourakis MJ, Martindale MQ (1996). Isolation, characterization, and expression of Le-msx, a maternally expressed member of the msx gene family from the glossiphoniid leech. Helobdella Dev Dyn.

[124] Isshiki T, Takeichi M, Nose A. The role of the msh homeobox gene during Drosophila neurogenesis: Implication for the dorsoventral specification of the neuroectoderm. Development. 1997;124:3099–109. 10.1242/dev.124.16.3099 9272951

[125] Ramos C, Robert B. msh/Msx gene family in neural development. Trends Genet. 2005;21(11):624–32. 10.1016/j.tig.2005.09.001 16169630

[126] Ma L, Swalla BJ, Zhou J, Dobias SL, Bell JR, Chen J (1996). Expression of an Msx homeobox gene in ascidians: Insights into the archetypal chordate expression pattern. Dev Dyn.

[127] Shimeld SM, McKay IJ, Sharpe PT (1996). The murine homeobox gene Msx-3 shows highly restricted expression in the developing neural tube. Mech Dev.

[128] Galle S, Yanze N, Seipel K (2005). The homeobox gene Msx in development and transdifferentiation of jellyfish striated muscle. Int J Dev Biol.

[129] Nose A, Isshiki T, Takeichi M (1998). Regional specification of muscle progenitors in Drosophila: The role of the msh homeobox gene. Development.

[130] Bendall AJ, Abate-Shen C. Roles for Msx and Dlx homeoproteins in vertebrate development. Gene. 2000;247(1-2):17–31. 10.1016/s0378-1119(00)00081-0 10773441

[131] Osumi N, Hirota A, Ohuchi H, Nakafuku M, Iimura T, Kuratani S (1997). Pax-6 is involved in the specification of hindbrain motor neuron subtype. Development.

[132] Ericson J, Rashbass P, Schedl A, Brenner-Morton S, Kawakami A, Van Heyningen V (1997). Pax6 controls progenitor cell identity and neuronal fate in response to graded Shh signaling. Cell.

[133] Florey E, Winesdorfer J (1968). Cholinergic nerve endings in octopus brain. J Neurochem.

[134] Dichmann DS, Harland RM (2011). Nkx6 genes pattern the frog neural plate and Nkx6.1 is necessary for motoneuron axon projection. Dev Biol..

[135] Saha MS, Miles RR, Grainger RM (1997). Dorsal-ventral patterning during neural induction in Xenopus: Assessment of spinal cord regionalization with xHB9, a marker for the motor neuron region. Dev Biol.

[136] Urbach R, Jussen D, Technau GM (2016). Gene expression profiles uncover individual identities of gnathal neuroblasts and serial homologies in the embryonic CNS of Drosophila. Development..

[137] Urbach R, Technau GM. Dorsoventral patterning of the brain: A comparative approach. Adv Exp Med Biol. 2008;628:42–56. 10.1007/978-0-387-78261-4_3 18683637

[138] Vergara HM, Bertucci PY, Hantz P, Tosches MA, Achim K, Vopalensky P (2017). Whole-organism cellular gene-expression atlas reveals conserved cell types in the ventral nerve cord of Platynereis dumerilii. Proc Natl Acad Sci U S A.

[139] Lauri A, Brunet T, Handberg-Thorsager M, Fischer AHL, Simakov O, Steinmetz PRH (2014). Development of the Annelid Axochord: Insights into notochord evolution. Science (80-)..

