# Supplementary material for: Unfolding the ventral nerve center of chaetognaths
Source: Neural Dev. 2024 May 8;19:5. doi: 10.1186/s13064-024-00182-6 (PMC11078758; doi:10.1186/s13064-024-00182-6)
Supplement: Supplementary file 3 — Additional file 3: Figure S1–7. Phylogenetic trees of genes of interest based on bilaterian protein sequences obtained from published literature and BLAST searches of the NCBI GenBank [file 13064_2024_182_MOESM3_ESM.pdf]

Supplementary figures

Unfolding the ventral nerve center of chaetognaths

June F. Ordoñez, Tim Wollesen

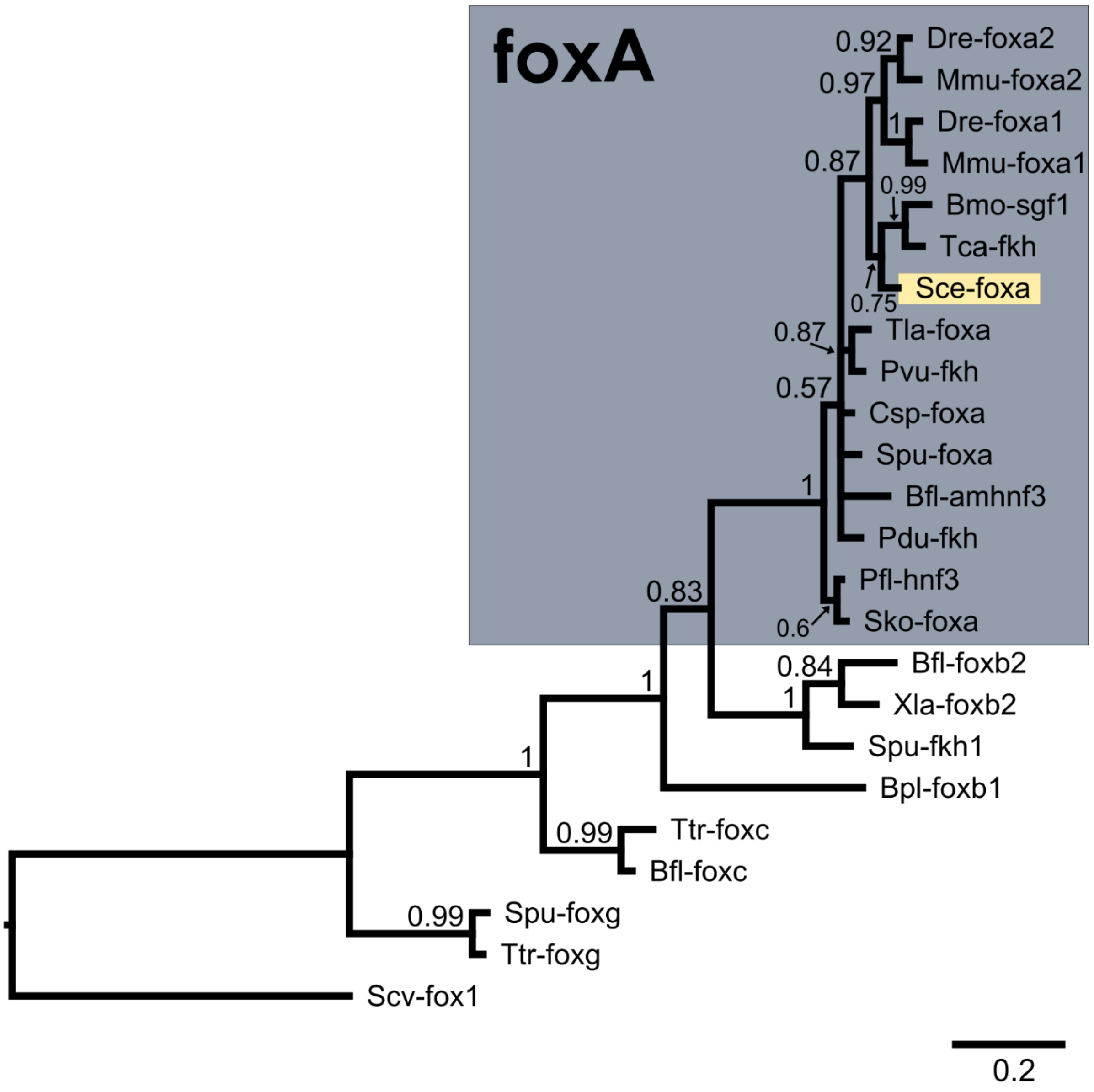

Figure S1. Phylogenetic tree of *fox* genes based on bilaterian protein sequences obtained from published literature and BLAST searches of the NCBI GenBank. The tree was generated using Bayesian likelihood analysis implemented in MrBayes plugin in Geneious Prime with the following configurations: LG + G was selected based on Protest 3.4, with four independent runs of 2,000,000 generations sampled every 100 generations and four chains each, and a burn-in length of 500,000. The support values of branches indicate posterior probabilities of Bayesian likelihood and rooted with *Saccharomyces cerevisiae fox1* (forkhead homeobox 1) gene as an outgroup. The foxA group is highlighted in the blue box and *Sce-foxA* in yellow. Species abbreviations are in Table S1.

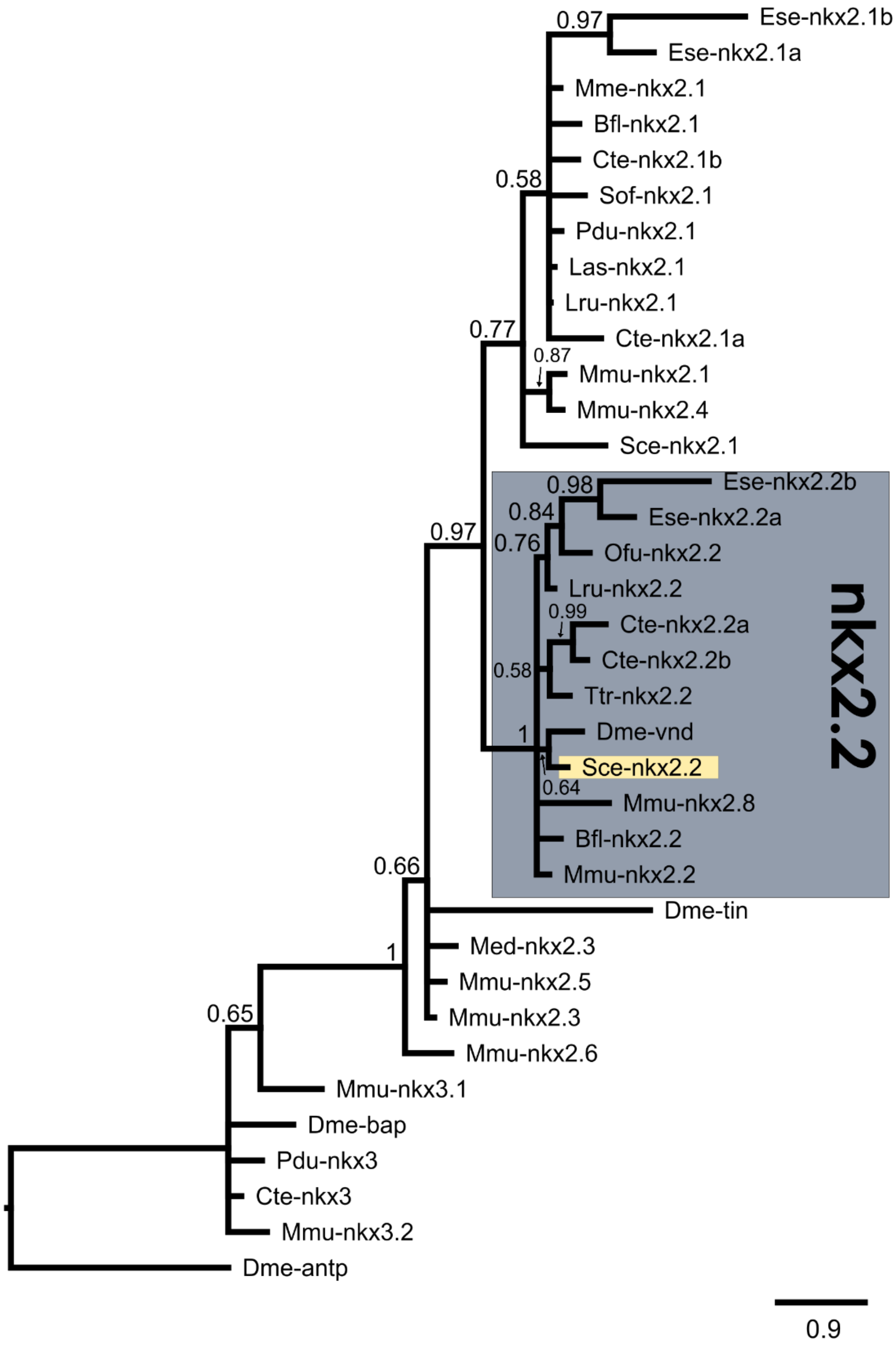

Figure S2. Phylogenetic tree of *nkx2* genes based on bilaterian protein sequences obtained from published literature and BLAST searches of the NCBI GenBank. The tree was generated using Bayesian likelihood analysis implemented in MrBayes plugin in Geneious Prime with the following configurations: LG + G was selected based on Protest 3.4, with four independent runs of 2,000,000 generations sampled every 100 generations and four chains each, and a burn-in length of 500,000. The support values of branches indicate posterior probabilities of Bayesian likelihood and rooted with *Drosophila melanogaster antp* (antennapedia) gene as an outgroup. The nkx2.2 group is highlighted in the blue box and Sce-nkx2.2 in yellow. Species abbreviations are in Table S1.

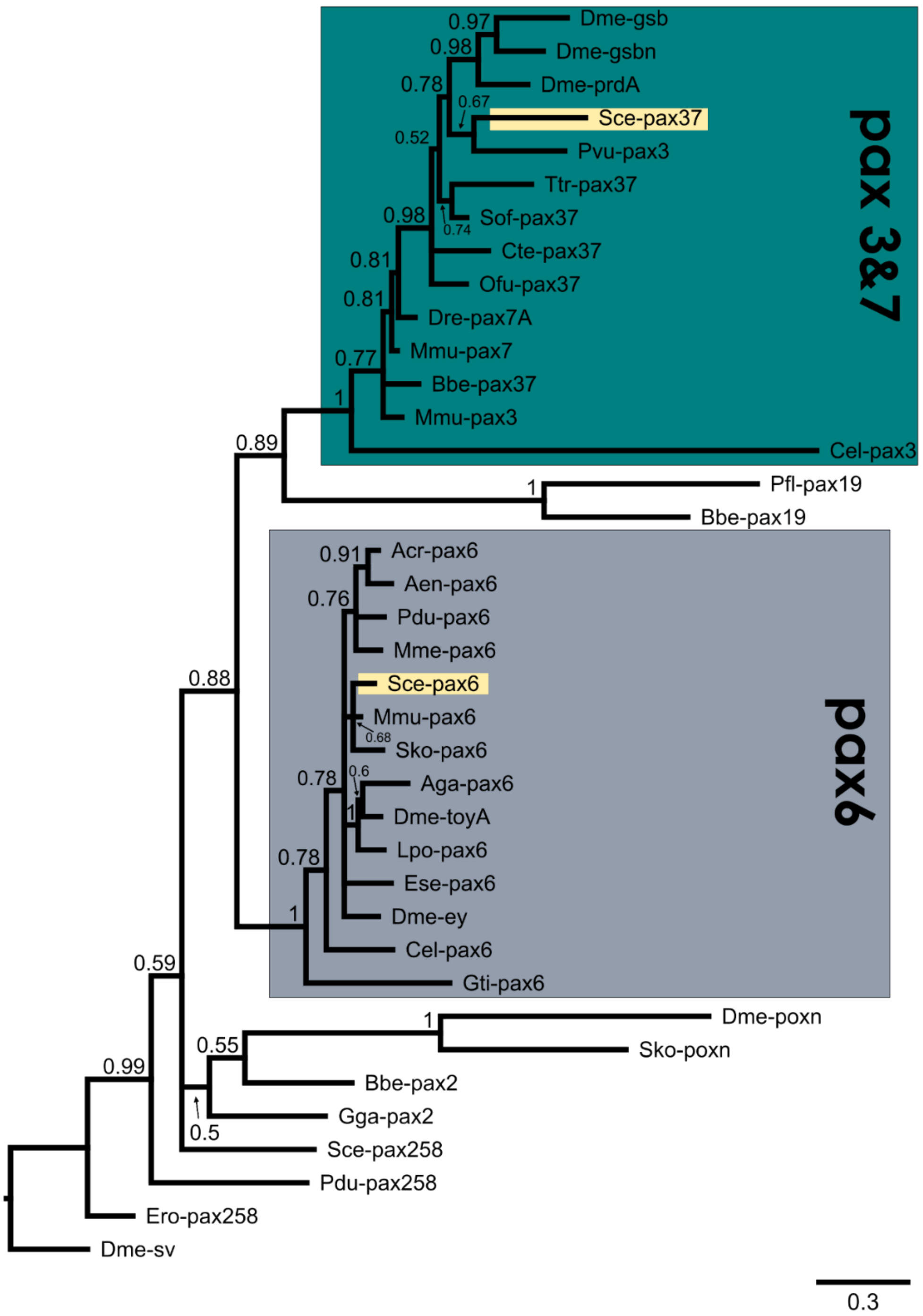

Figure S3. Phylogenetic tree of *pax* genes based on bilaterian protein sequences obtained from published literature and BLAST searches of the NCBI GenBank. The tree was generated using Bayesian likelihood analysis implemented in MrBayes plugin in Geneious Prime with the following configurations: VT + I + G was selected based on Protest 3.4, with four independent runs of 2,000,000 generations sampled every 100 generations and four chains each, and a burn-in length of 500,000. The support values of branches indicate posterior probabilities of Bayesian likelihood and rooted with *Drosophila melanogaster sv* (shaven) gene as an outgroup. The pax3/7 group is indicated by the teal box and while the pax6 is in the blue box. *Sce-pax3/7* and *Sce-pax6* are highlighted in yellow. Species abbreviations are in Table S1.

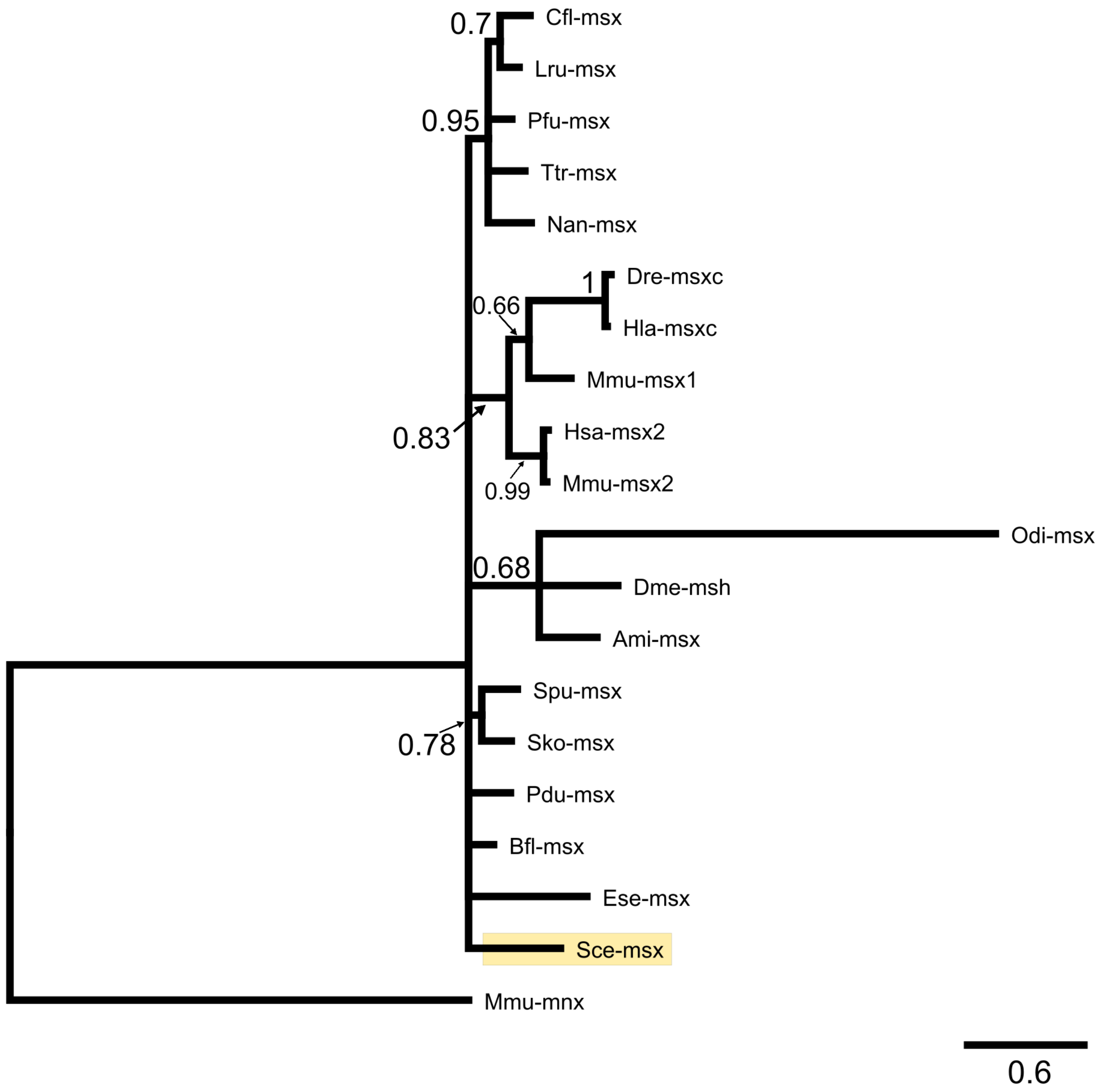

Figure S4. Phylogenetic tree of *msx* genes based on bilaterian protein sequences obtained from published literature and BLAST searches of the NCBI GenBank. The tree was generated using Bayesian likelihood analysis implemented in MrBayes plugin in Geneious Prime with the following configurations: JTT + G was selected based on Protest 3.4, with four independent runs of 2,000,000 generations sampled every 100 generations and four chains each, and a burn-in length of 500,000. The support values of branches indicate posterior probabilities of Bayesian likelihood and rooted with *Mus musculus mnx* (motor neuron and pancreas homeobox 1) gene as an outgroup. *Sce-msx* is highlighted in yellow. Species abbreviations are in Table S1.

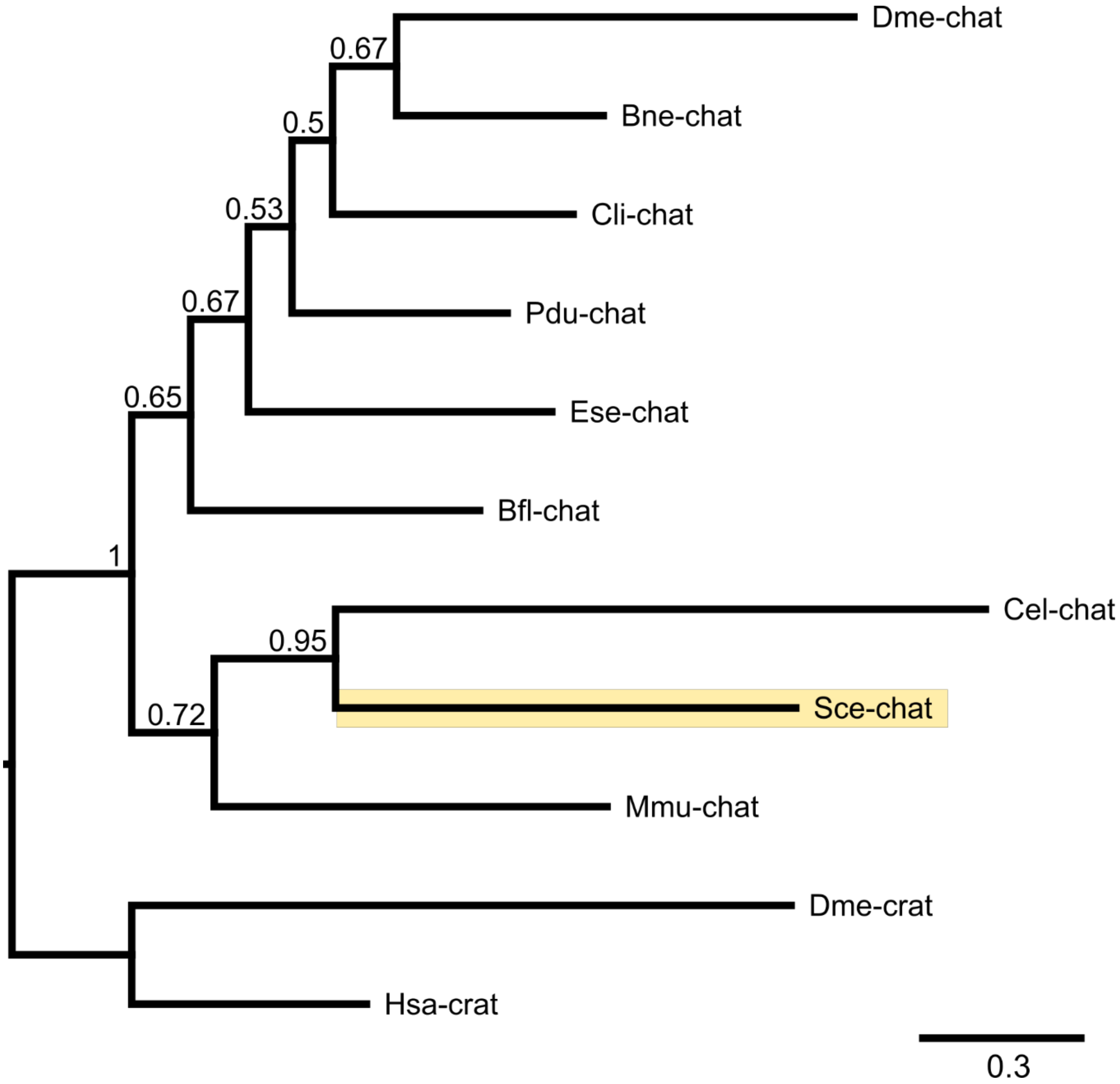

Figure S5. Phylogenetic tree of *ChAT* genes based on bilaterian protein sequences obtained from published literature and BLAST searches of the NCBI GenBank. The tree was generated using Bayesian likelihood analysis implemented in MrBayes plugin in Geneious Prime with the following configurations: LG + I +G was selected based on Protest 3.4, with four independent runs of 2,000,000 generations sampled every 100 generations and four chains each, and a burn-in length of 500,000. The support values of branches indicate posterior probabilities of Bayesian likelihood and rooted with *Drosophila melanogaster and Homo sapiens crat* (carnitine o-acetyltransferase) gene as an outgroup. *Sce-ChAT* is highlighted in yellow. Species abbreviations are in Table S1.

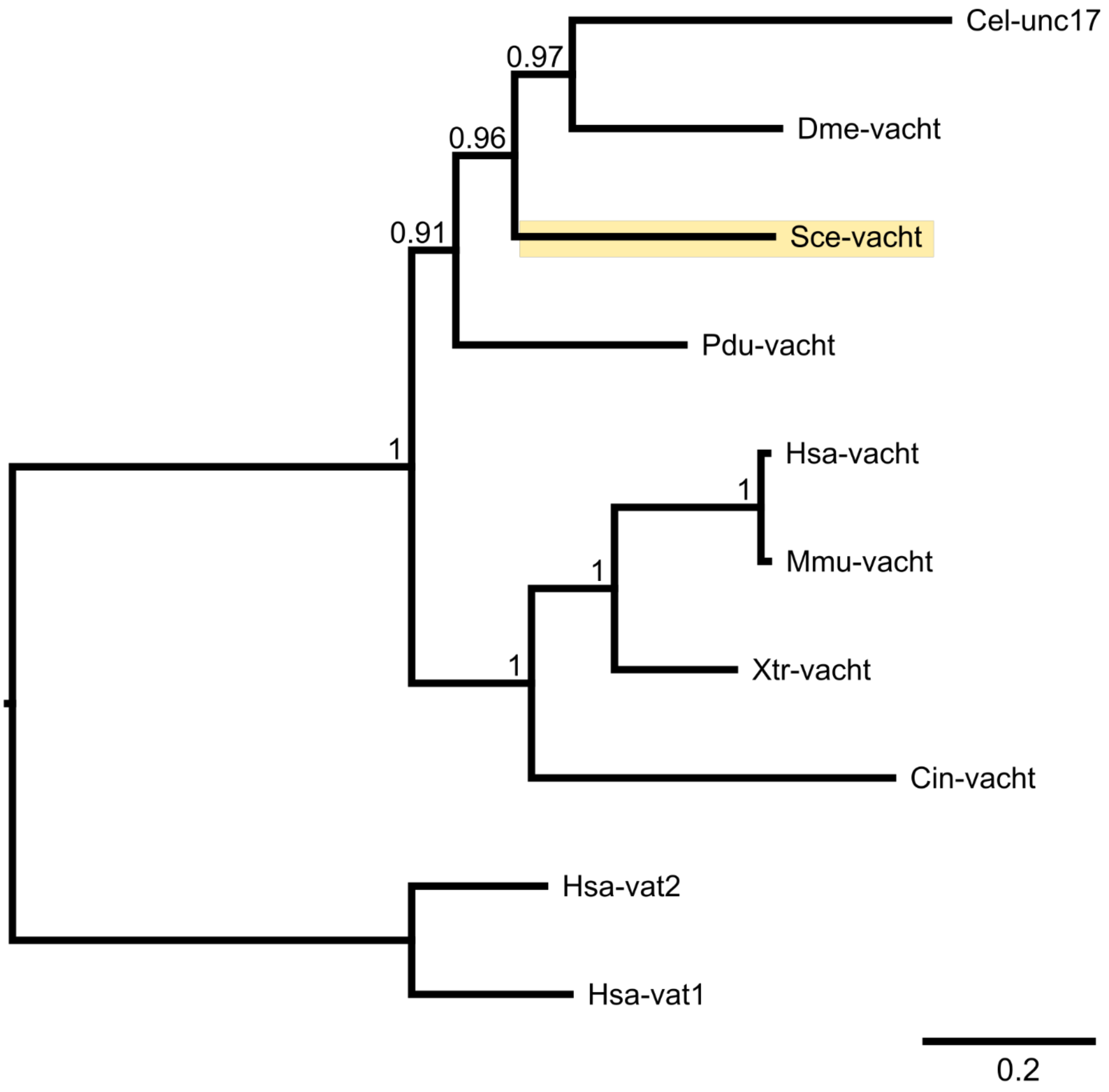

Figure S6. Phylogenetic tree of *VAChT* genes based on bilaterian protein sequences obtained from published literature and BLAST searches of the NCBI GenBank. The tree was generated using Bayesian likelihood analysis implemented in MrBayes plugin in Geneious Prime with the following configurations: JTT + G was selected based on Protest 3.4, with four independent runs of 2,000,000 generations sampled every 100 generations and four chains each, and a burn-in length of 500,000. The support values of branches indicate posterior probabilities of Bayesian likelihood and rooted with *Homo sapiens vat1* and *vat2* (vesicular amine transporter 1 and 2) genes as outgroups. *Sce-VAChT* is highlighted in yellow. Species abbreviations are in Table S1.

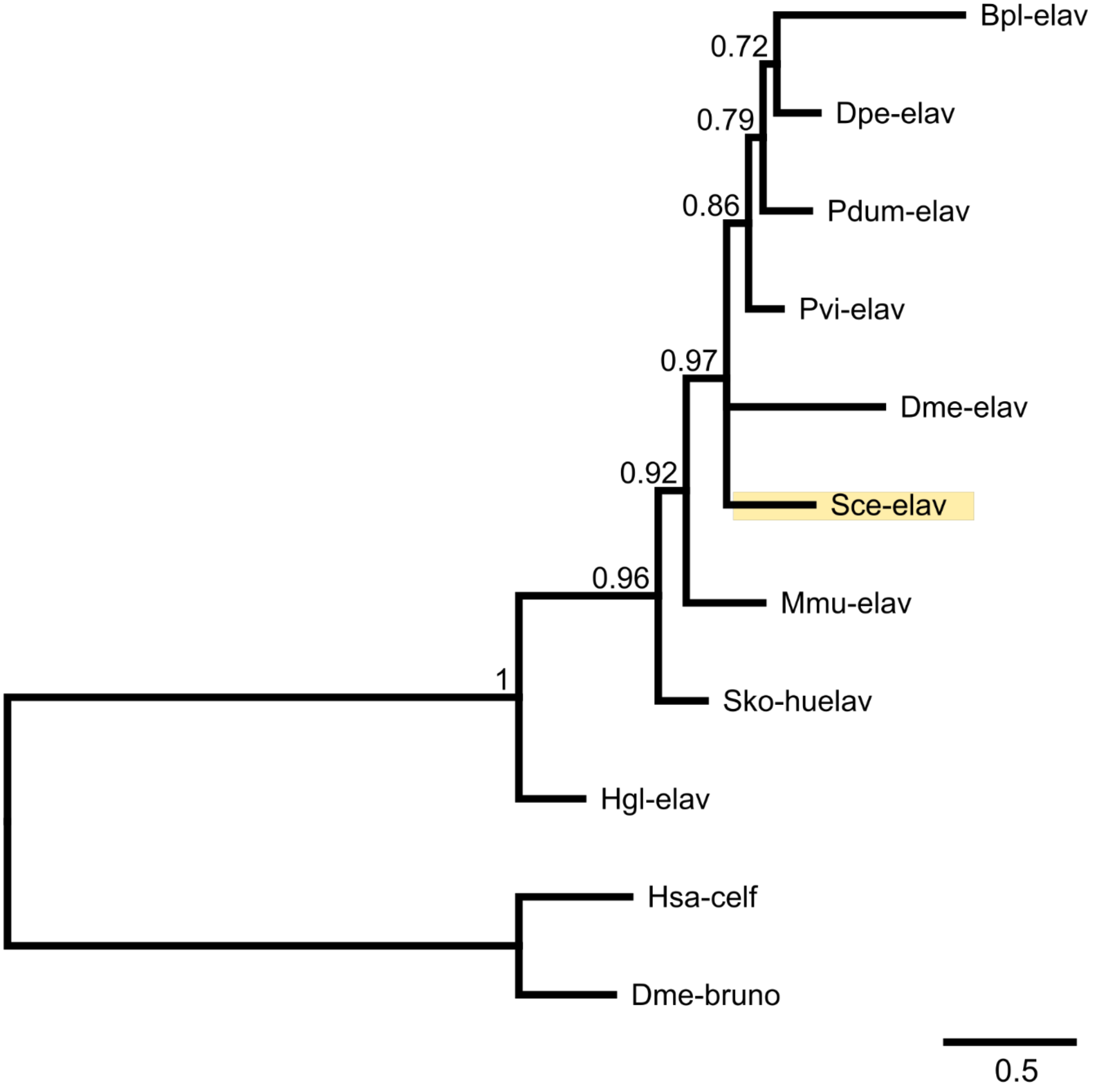

Figure S7. Phylogenetic tree of *elav* genes based on bilaterian protein sequences obtained from published literature and BLAST searches of the NCBI GenBank. The tree was generated using Bayesian likelihood analysis implemented in MrBayes plugin in Geneious Prime with the following configurations: LG + G +F was selected based on Protest 3.4, with four independent runs of 2,000,000 generations sampled every 100 generations and four chains each, and a burn-in length of 500,000. The support values of branches indicate posterior probabilities of Bayesian likelihood and rooted with *Drosophila melanogaster bruno* and *Homo sapiens celf* (CUGBP Elav-Like Family) genes as outgroups. *Sce-elav* is highlighted in yellow. Species abbreviations are in Table S1.
