# Supplementary figures and images for: Unfolding the ventral nerve center of chaetognaths
Source: Neural Dev. 2024 May 8;19:5. doi: 10.1186/s13064-024-00182-6 (PMC11078758; doi:10.1186/s13064-024-00182-6)

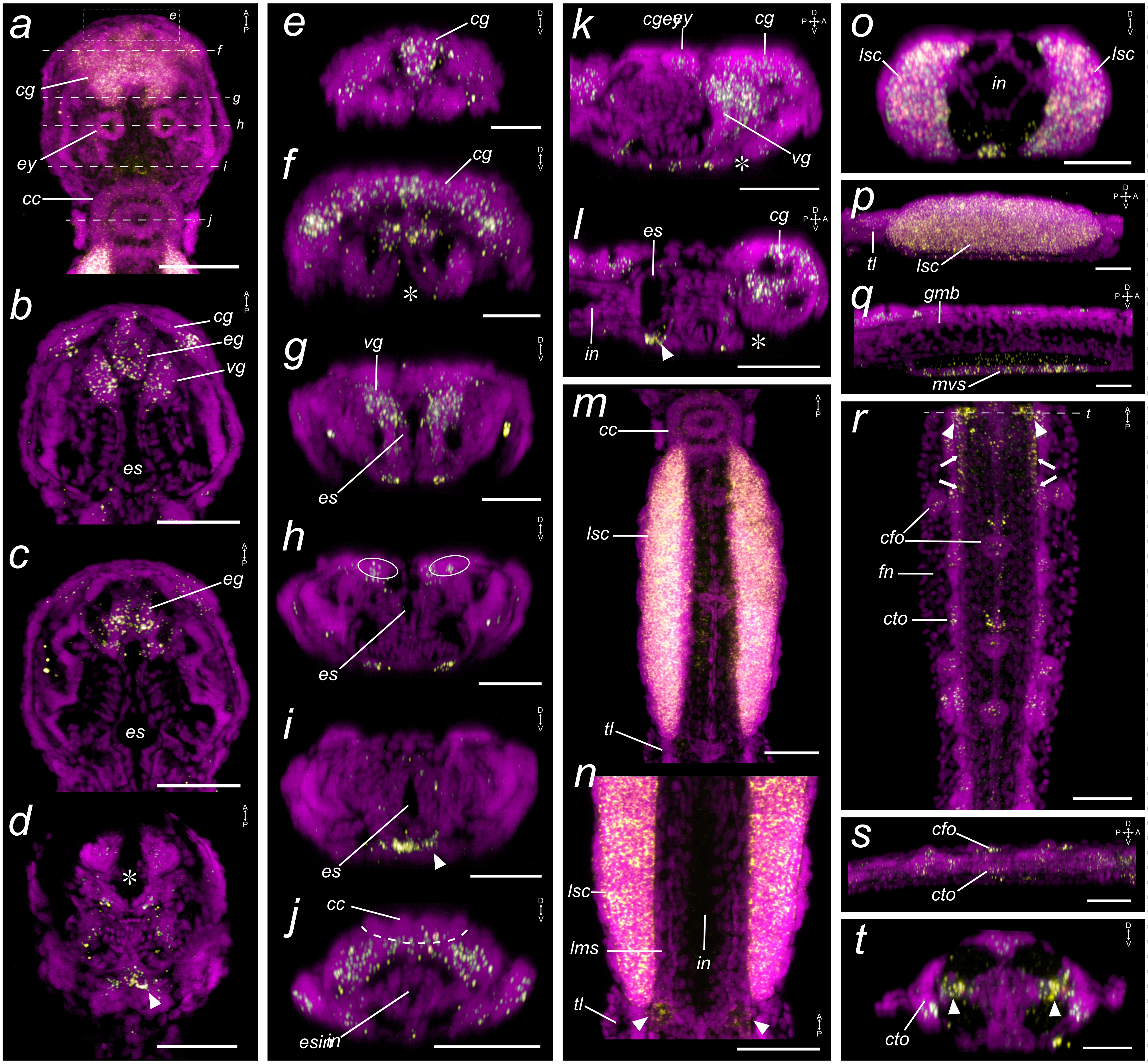

Supplement: Supplementary file 4 — Additional file 4: Figure S8. Expression patterns of elav in an early juvenile (7–10 dph) of S. cephaloptera. Gene transcripts are visualized with AP-Fast Blue (yellow) and cell nuclei with DAPI (purple). (a– d) Horizontal confocal sections of the head from (a) dorsal to (d) ventral. (d) An expression domain is detected lining the ventral esophagus, which is the location of the presumed sub-esophageal ganglion (arrowhead; also shown in panels i and l). (e—j) Transverse profiles of the head, from (e) anterior to (j) posterior. (h) Ellipses indicate the location of the eyes. Transverse section locations are marked in a. (k, l) Lateral profiles of the head along the (k) eye, exposing the lateral view of the vestibular ganglion and (l) longitudinal midline of the head. (m) Maximum projection of the entire trunk. (n) A medio-dorsal profile of (m) showing the longitudinal muscle cells and posterior terminus of the intestine. (o) Transverse profile of the trunk. (p) Lateral maximum projection of the trunk. (q) Lateral profile along the midline of trunk). (r) Maximum horizontal and (s) lateral projections of the tail. An expression domain (arrowhead) is detected in the anterior-most tail where the presumptive germ cells are developing (also shown in panels n and t) and in the lateral cells posterior to it (arrows). (t) Transverse profile of the anterior tail (refer to r for the section location). Scale bars: 50 μm, except panels (p) and (q) (100 μm). Asterisk indicates the position of the mouth opening. Orientation of specimens is indicated in the top right corner of each panel. cc, corona ciliata; cfo, ciliary fence organ; cg, cerebral ganglion; cp, cephalic adhesive papillae; cto, ciliary tuft organ; dlm, dorsal longitudinal muscle; es, esophagus; eg, esophageal ganglion; ey, eye; fn, fin; gmb, gut-muscle bundle; in, intestine; lsc, lateral somata clusters; mvs, medioventral somata clusters; tl, tail; vg, vestibular ganglion; vlm, ventral longitudinal muscle. [file 13064_2024_182_MOESM4_ESM.tif]

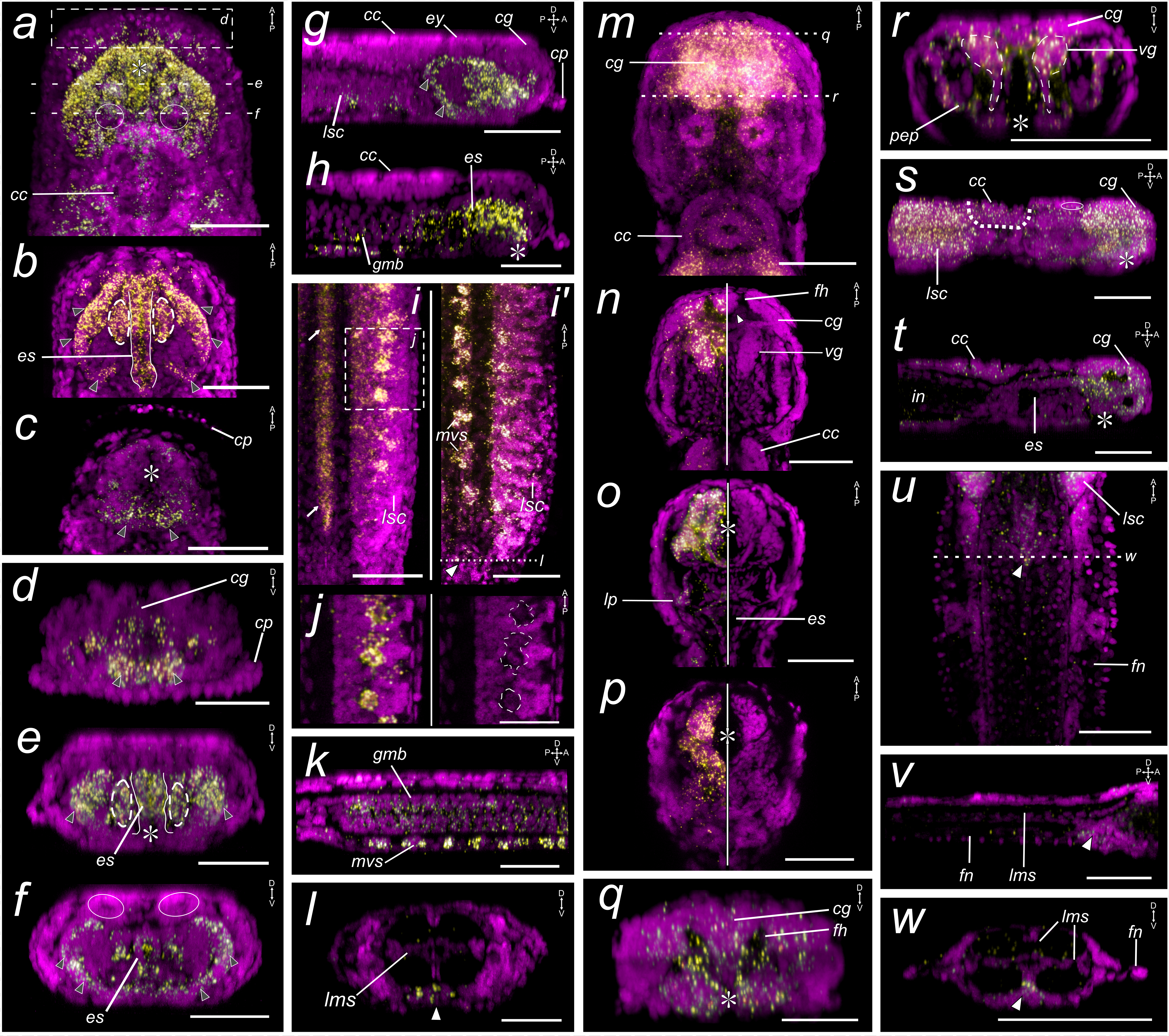

Supplement: Supplementary file 5 — Additional file 5: Figure S9. Expression patterns of foxA in hatchlings (1 dph) and early juveniles (7–10 dph) of S. cephaloptera. Gene transcripts are visualized with AP-Fast Blue (yellow) and cell nuclei with DAPI (purple). (a – l) Confocal sections of a hatchling. (a) Horizontal maximum projection of the head. Eyes are encircled. (b) Mid head horizontal profile showing that Sce-foxA expression is prominent in cells arranged in a bulbous shape (red arrowheads, also visualized in c—g), cells lining the mouth and esophagus (solid outline), and in two cell clusters flanking the esophagus (dashed oval outline). (c) Ventral horizontal confocal section of the head. (d – f) Transverse profiles of the head, from (d) anterior to (f) posterior. (f) Eye locations are encircled. Transverse section locations are marked in a. (g, h) Lateral profiles of the head along the (g) eye and (h) midline of the head. (i, i') In the trunk, Sce-foxA expression domains are detected in the intestine (i, k; arrows), in lateral (i, i', j) and medioventral somata clusters (i', k), and in the ventroposterior trunk (l). (j) Higher magnification of the Sce-foxA+ large neuronal cells (left panel) and the DAPI channel with the expression boundary in dashed outline (right panel). (k) Lateral profile along the midline of gut-muscle bundle. (l) Transverse profile of the posterior trunk showing expression in ventral cells (arrowhead). Section location is indicated in i’. (m – w) Section profiles of an early juvenile. (m – p) Horizontal confocal sections of the head. Intense expression is observed in the (m) cerebral ganglion, (n) vestibular ganglia, and (o, p) cells around the mouth area. In n – p, the right panels only show the DAPI channel. (q) Transverse profiles of the anterior and (r) posterior cerebral ganglion. Vestibular ganglia are indicated in dashed outline. (s, t) Lateral profiles of the head. (s) Lateral projection or the whole head. (t) Lateral view along the longitudinal midline of the he [file 13064_2024_182_MOESM5_ESM.tif]

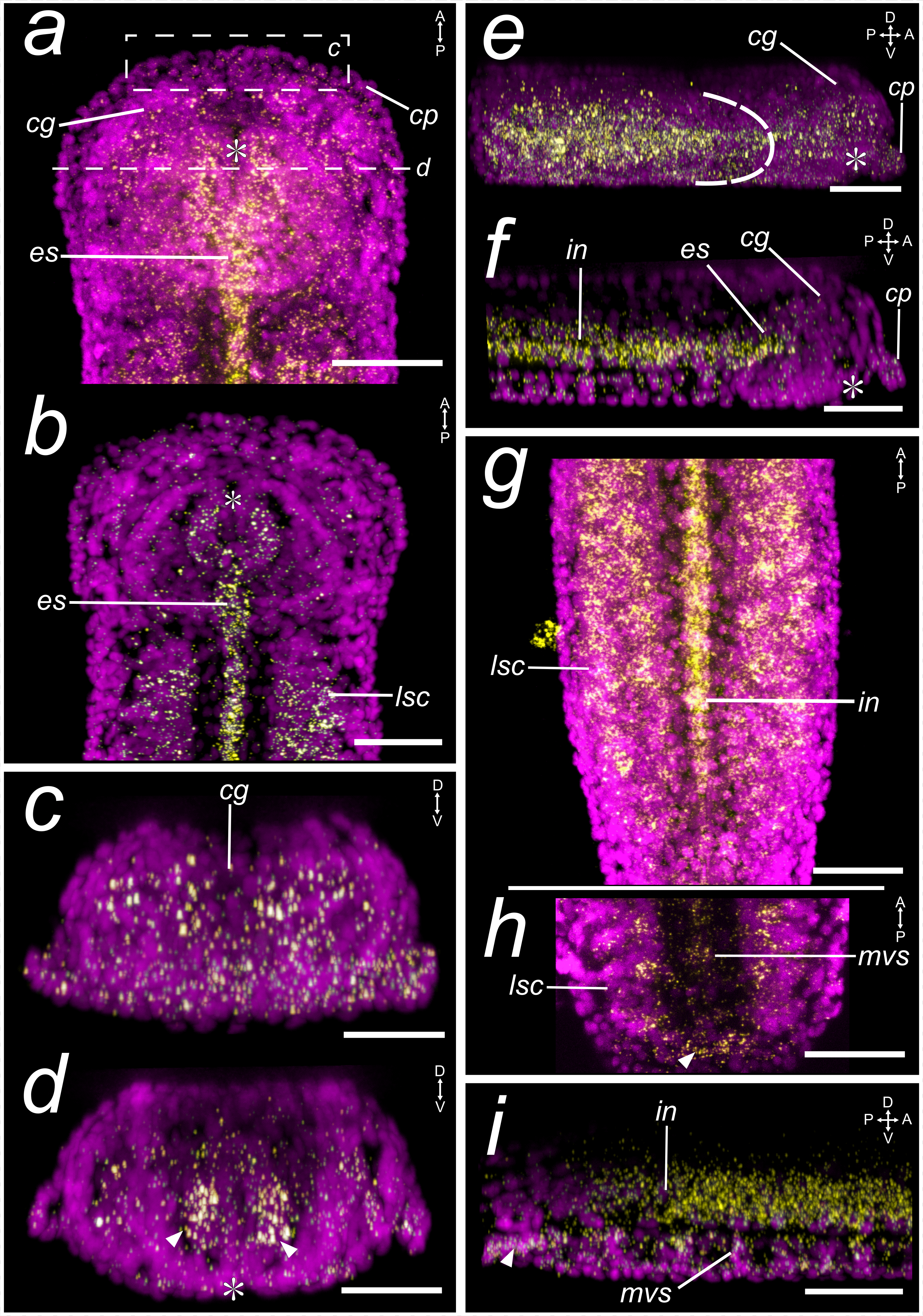

Supplement: Supplementary file 6 — Additional file 6: Figure S10. Expression patterns of Sce-nkx2.2 in S. cephaloptera hatchlings. Gene transcripts are visualized with AP-Fast Blue (yellow) and cell nuclei with DAPI (purple). (a) Horizontal maximum projection (ventral view) and (b) mid horizontal profile of the head. (c, d) Transverse profiles of the (c) anterior head and (d) along the future mouth (asterisk). (e, f) Lateral profiles of the (e) entire anterior half and along the midline exposing the expression in the (f) gut. (g) Horizontal maximum projection of the posterior trunk. (h) Ventral horizontal profile in the posterior trunk and (i) lateral profile along the trunk midline showing expression in mvs and cells in the ventroposterior lateral somata cluster (arrowhead). Scale bars: 50 μm. The asterisk indicates the position of the mouth opening. Orientation of specimens is in the top right corner of each panel. cg, cerebral ganglion; cp, cephalic adhesive papillae; es, esophagus; in, intestine; lsc, lateral somata clusters; mvs, medioventral somata clusters. [file 13064_2024_182_MOESM6_ESM.tif]

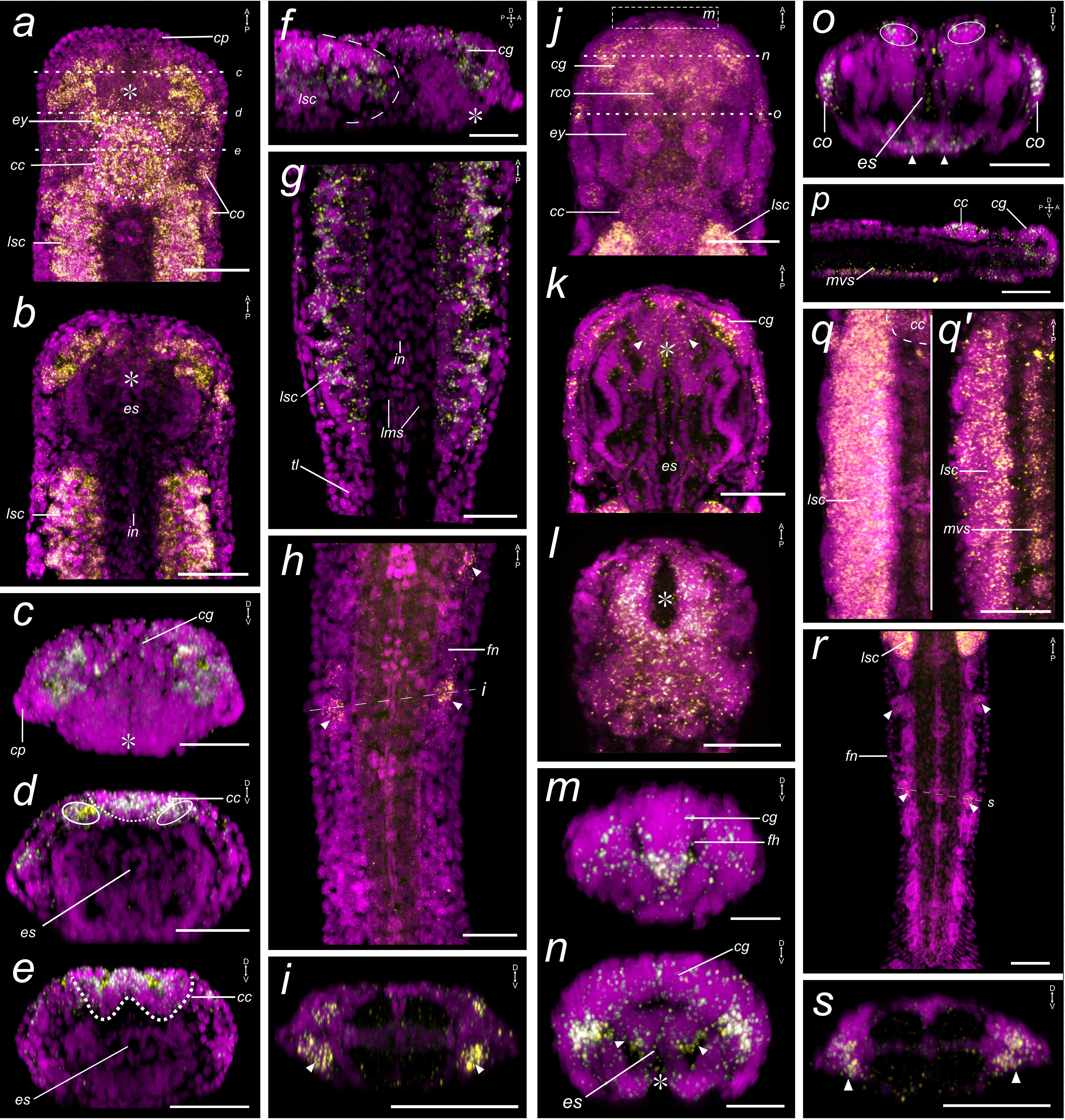

Supplement: Supplementary file 7 — Additional file 7: Figure S11. Expression patterns of Sce-pax6 in hatchlings (1 dph) and early juveniles (7–10 dph) of S. cephaloptera. Gene transcripts are visualized with AP-Fast Blue (yellow) and nuclei with DAPI (purple). (a – i) Section profiles of a hatchling. (a) Maximum horizontal projection of the anterior body. (b) Horizontal profile along the midplane of the anterior body. (c – e) Transverse profiles of the head. The location of the eyes is encircled and the corona ciliata is bordered by a dashed outline. (f) Lateral profile of the anterior body. Dashed outline indicates the anterior boundary of the lateral somata clusters. (g) Horizontal profile along the midplane of the trunk. (h) Horizontal maximum projection of the tail showing expression domains in sensory organs (arrowheads). (i) Transverse profile of the sensory organs on the lateral tail. Location of the section is indicated in h. (j – s) Section profiles of an early juvenile. (j) Maximum horizontal projection of the head. (k, l) Horizontal profiles along the (k) mid and (l) ventral plane. (k, n) The presumptive esophageal ganglia show a slight Sce-pax6 expression (arrowheads). (m – o) Transverse profiles of the head. (p) Lateral profile along the midline of the anterior body. (q) Horizontal maximum projection of the half of the trunk and (q’) horizontal profile of the ventral plane of q. (r) Horizontal maximum projection of the tail with ciliary fence receptors in arrows. (s) Transverse profile along the ciliary fence organ of the tail (refer to r for the section location). Scale bars: 50 μm. The asterisk indicates the position of the mouth opening. Orientation of specimens is in the top right corner of each panel. cc, corona ciliata; co, ciliary organ; cg, cerebral ganglion; cp, cephalic adhesive papillae; es, esophagus; ey, eye; fh: frontal horn; fn, fin; gmb, gut-muscle bundle; in, intestine; lsc, lateral somata clusters; lms, longitudinal muscle somata; mvs, medioventral somata clusters; rco: [file 13064_2024_182_MOESM7_ESM.tif]

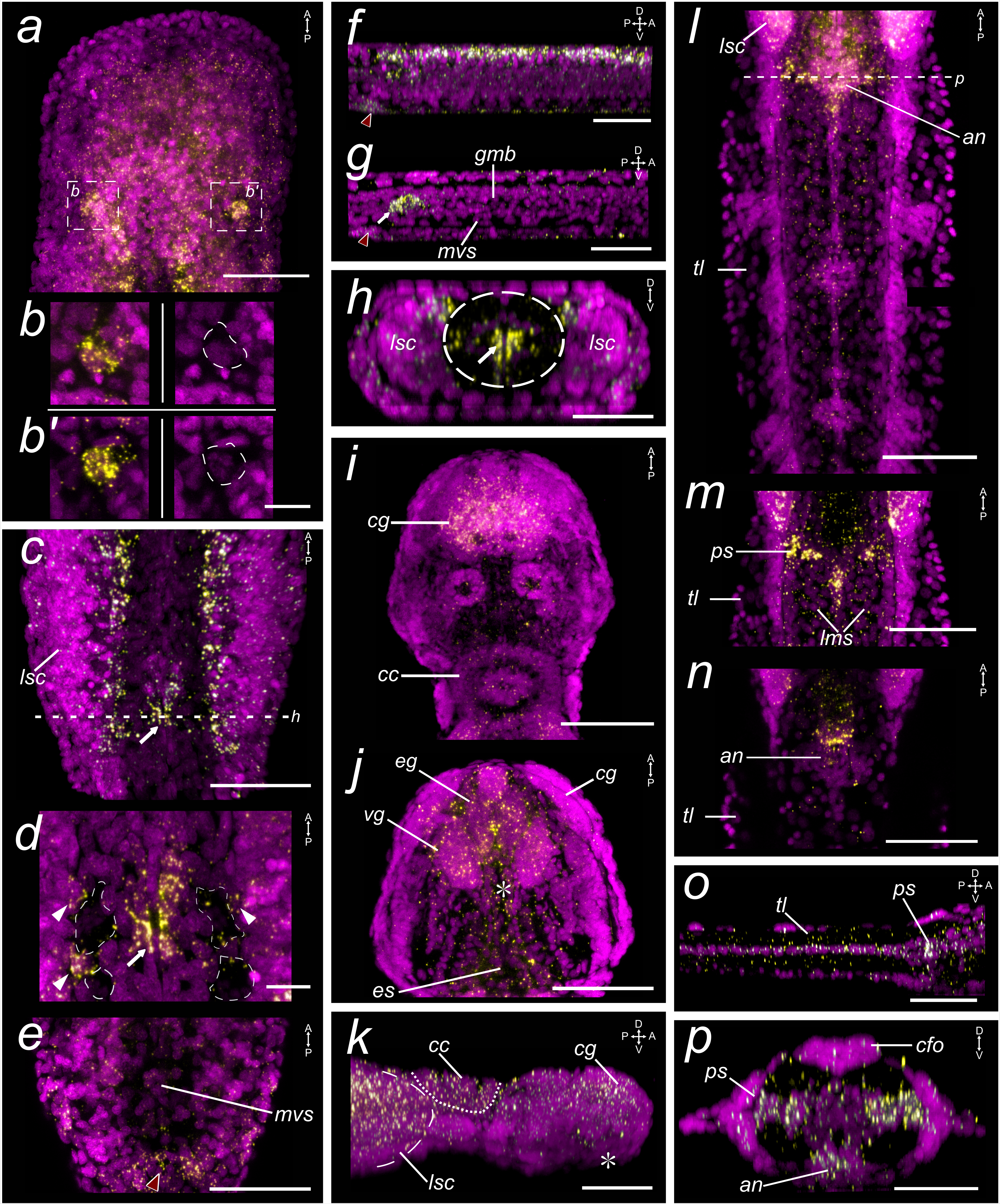

Supplement: Supplementary file 8 — Additional file 8: Figure S12. Expression patterns of Sce-pax3/7 in hatchlings (1 dph) and early juveniles (7–10 dph) of S. cephaloptera. Gene transcripts are visualized with AP-Fast Blue (yellow) and cell nuclei with DAPI (purple). (a – h) Section profiles of a hatchling. (a) Horizontal maximum projection of the head. (b, b’) Higher magnification of the two Sce-pax3/7+ cells in the anterior terminal of lateral somata clusters (left panels) and the DAPI channel with the expression boundary in dashed outline (right panels). (c – e) Horizontal confocal sections of the trunk-tail boundary. (d) Magnified view of the horizontal midline showing expression in specialized mesodermal cells (presumptive peri-intestinal cells in arrow and lateral cells in arrowheads) separating the germ cells (dashed outline). (e) Expression domain in the ventral cells (red arrowhead) posterior to the medioventral somata clusters. (f, g) Lateral profile of the trunk along the (f) lateral somata clusters exhibiting the longitudinal expression pattern and (g) longitudinal midline (gut-muscle bundle) showing the Sce-pax3/7+ cells in the posterior terminal of the gut. (h) Transverse profile of the posterior trunk (section location in c). Encircled is the gut-muscle bundle. (i – p) Section profiles of a juvenile. (i, j) Horizontal profiles of the head showing expression in the cephalic ganglia. (k) Lateral view of the whole head. (l) Horizontal maximum projection of the tail. (m, n) Horizontal profiles of the trunk-tail boundary showing the expression domains in (m) posterior septum in mid-section and (n) anus in the ventral plane. (o) Lateral profile along the tail longitudinal midline. (p) Transverse profile along the posterior septum and anal region. Scale bars: 50 μm, except panels b, b’, d (15 μm). The asterisk indicates the position of the mouth opening. Orientation of specimens is in the top right corner of each panel. an, anus; cc, corona ciliata; cfo, ciliary fence organ; cg, cerebral gang [file 13064_2024_182_MOESM8_ESM.tif]

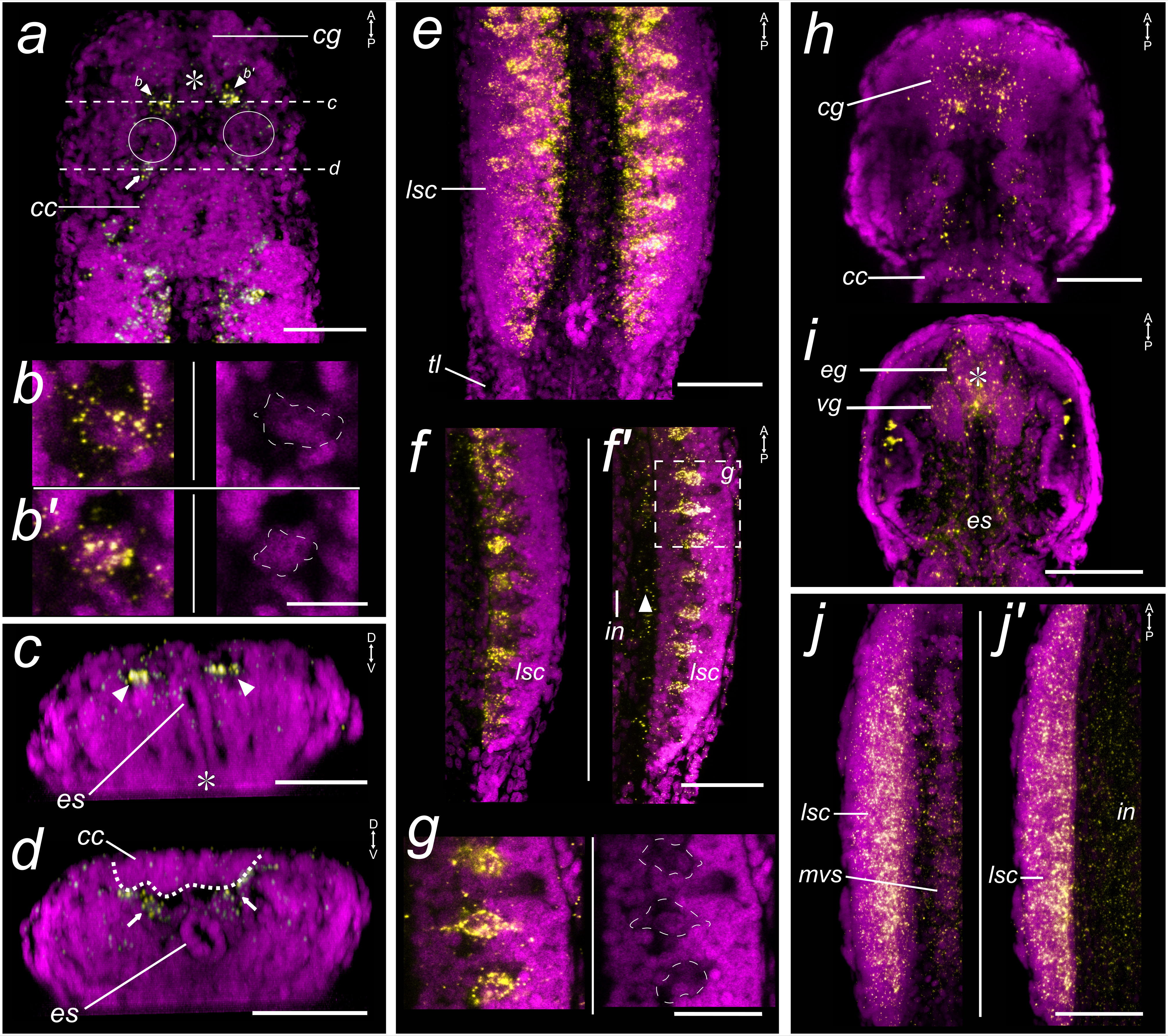

Supplement: Supplementary file 9 — Additional file 9: Figure S13. Expression patterns of msx in hatchlings (1 dph) and juveniles (7–10 dph) of S. cephaloptera. Gene transcripts are visualized with AP-Fast Blue (yellow) and cell nuclei with DAPI (purple). (a – g) Section profiles of a hatchling. (a) Horizontal maximum projection of the head. Eye is encircled. (b, b’) Higher magnification of the two Sce-msx+ cells in the head (left panels) and the DAPI channel with the expression boundary in dashed outline (right panels). (c) Cells in b and b’ (arrowheads) in transverse view. (d) Transverse profile showing expression in cells in the anteroventral corona ciliata (arrows). (e) Horizontal maximum projection of the trunk. (f, f’) Horizontal profiles of the midtrunk showing Sce-msx signal in large neuronal cells of the lateral somata clusters and in muscle fibers (arrowheads). f is a dorsal section, while f’ is along the midplane of the intestine. (g) Higher magnification of the Sce-msx+ large neuronal cells (left panel) and the DAPI channel with the expression boundary in dashed outline (right panel). (h – j) Section profiles of a juvenile. (h, i) Horizontal profiles of the head. Expression is more prominent in the (h) posterior cerebral ganglion and in the (i) vestibular and esophageal ganglia. (j) Maximum horizontal projection of the trunk and (j’) horizontal profile of the mid trunk. Scale bars: 50 μm, except panels b, b’, and g (15 μm). Asterisk indicates the position of the mouth opening. Orientation of specimens is indicated in the top right corner of each panel. cc, corona ciliata; cg, cerebral ganglion; es, esophagus; eg, esophageal ganglion; in, intestine; lsc, lateral somata clusters; mvs: medioventral somata clusters; tl: tail; vg, vestibular ganglion. [file 13064_2024_182_MOESM9_ESM.tif]

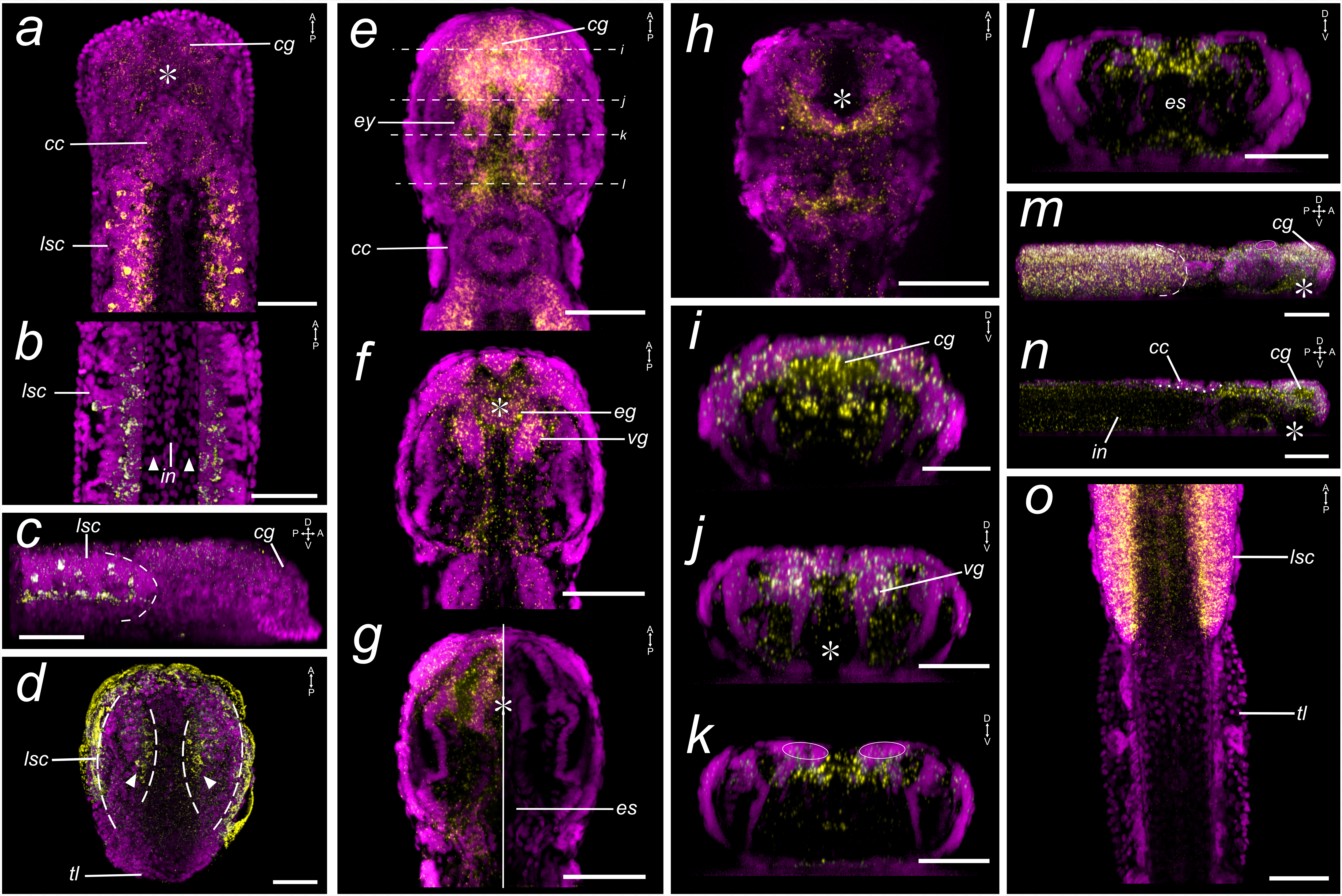

Supplement: Supplementary file 10 — Additional file 10: Figure S14. Expression patterns of Sce-ChAT in hatchlings (1 dph) and early juveniles (7–10 dph) of S. cephaloptera. Gene transcripts are visualized with AP-Fast Blue (yellow) and cell nuclei with DAPI (purple). (a-c) Section profiles of a hatchling. (a) Maximum horizontal projection of the anterior half of the body. (b) Horizontal profile of the trunk showing Sce-ChAT expression in lateral cell somata adjacent to the muscle cells (arrowheads). (c) Lateral profile of anterior half of the body. (d) Late-stage encapsulated embryo with signal (arrowheads) in medial lateral somata cluster (dashed outline). The egg capsule around the embryo is strongly stained. (e – o) Section profiles of a juvenile. (e) Maximum horizontal projection of the head. (f – h) Horizontal profiles of the head from dorsal (f) to ventral (h). (g) Expression pattern is shown on the left panel and DAPI-only channel on the right panel for a better representation of the expression extent. (i – l) Transverse profiles of the head from (i) anterior to (l) posterior. In (k), the location of the eyes is encircled. (m) Lateral profiles of the whole anterior half. (n) Lateral profile along the longitudinal midline of the anterior half. (o) Horizontal maximum projection of the posterior trunk and anterior tail. Scale bars: 50 μm. Asterisk indicates the position of the mouth opening. Orientation of specimens is indicated in the top right corner of each panel. cc, corona ciliata; cg, cerebral ganglion; es, esophagus; eg, esophageal ganglion; ey, eye; in, intestine; lsc, lateral somata clusters; tl, tail; vg, vestibular ganglion. [file 13064_2024_182_MOESM10_ESM.tif]

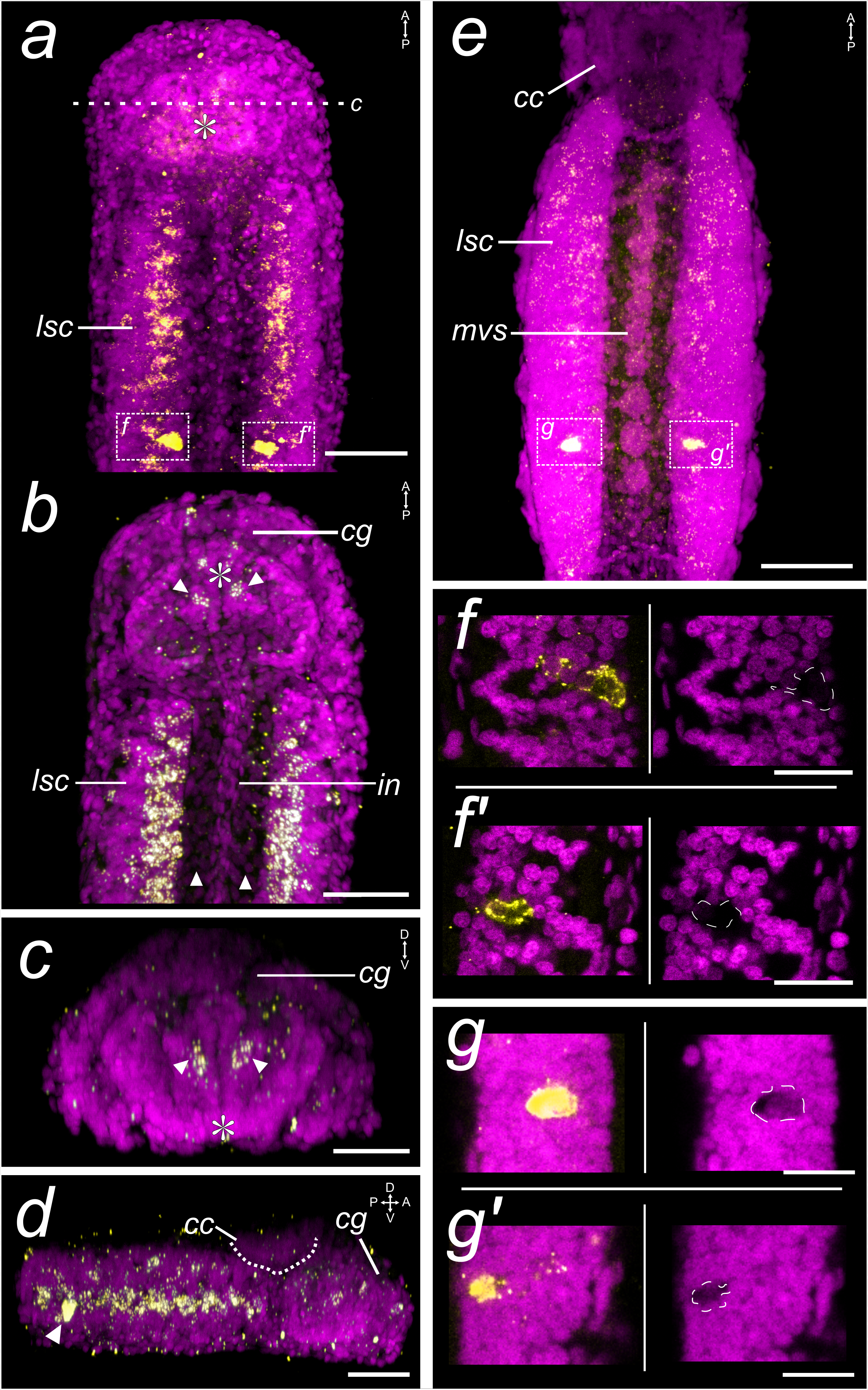

Supplement: Supplementary file 11 — Additional file 11: Figure S15. Expression patterns of Sce-VAChT in hatchlings (1 dph) and early juveniles (7–10 dph) of S. cephaloptera. Gene transcripts are visualized with AP-Fast Blue (yellow) and cell nuclei with DAPI (purple). (a – d, f, f’) Section profiles of a hatchling. (a) Maximum horizontal projection of the anterior half of the animal. (b) Horizontal profile of the trunk showing Sce-VAChT expression in lateral cell somata adjacent to the muscle cells (arrowheads). (c) Transverse profile of the head showing expression domains in cells flanking the oral cavity (dorsal to the asterisk). (d) Lateral profile of the anterior half. (e, g, g’) Section profiles of a juvenile. (e) Horizontal maximum projection of the trunk. (f, f’, g, g’) Higher magnification of the Sce-VAChT+ large neuronal cells (left panels) and the DAPI channel with the expression boundary in dashed outline (right panels) in hatchling and early juveniles, respectively. Scale bars: 50 μm, except panels f, f’, g, and g’ (15 μm). cc, corona ciliata; cg, cerebral ganglion; in, intestine; mvs, medioventral somata clusters; lsc, lateral somata clusters. [file 13064_2024_182_MOESM11_ESM.tif]
